# The human gut symbiont *Ruminococcus gnavus* shows specificity to blood group A antigen during mucin glycan foraging: Implication for niche colonisation in the gastrointestinal tract

**DOI:** 10.1371/journal.pbio.3001498

**Published:** 2021-12-22

**Authors:** Haiyang Wu, Emmanuelle H. Crost, C David Owen, Wouter van Bakel, Ana Martínez Gascueña, Dimitrios Latousakis, Thomas Hicks, Samuel Walpole, Paulina A. Urbanowicz, Didier Ndeh, Serena Monaco, Laura Sánchez Salom, Ryan Griffiths, Raven S. Reynolds, Anna Colvile, Daniel I. R. Spencer, Martin Walsh, Jesus Angulo, Nathalie Juge

**Affiliations:** 1 Quadram Institute Bioscience, Norwich, United Kingdom; 2 Diamond Light Source Ltd, Didcot, United Kingdom; 3 Research Complex at Harwell, Didcot, United Kingdom; 4 University of East Anglia, Norwich, United Kingdom; 5 Ludger Ltd, Abingdon, United Kingdom; 6 Universidad de Sevilla and Instituto de Investigaciones Químicas, Sevilla, Spain; UNITED STATES

## Abstract

The human gut symbiont *Ruminococcus gnavus* displays strain-specific repertoires of glycoside hydrolases (GHs) contributing to its spatial location in the gut. Sequence similarity network analysis identified strain-specific differences in blood-group endo-β-1,4-galactosidase belonging to the GH98 family. We determined the substrate and linkage specificities of GH98 from *R*. *gnavus* ATCC 29149, *Rg*GH98, against a range of defined oligosaccharides and glycoconjugates including mucin. We showed by HPAEC-PAD and LC-FD-MS/MS that *Rg*GH98 is specific for blood group A tetrasaccharide type II (BgA II). Isothermal titration calorimetry (ITC) and saturation transfer difference (STD) NMR confirmed *Rg*GH98 affinity for blood group A over blood group B and H antigens. The molecular basis of *Rg*GH98 strict specificity was further investigated using a combination of glycan microarrays, site-directed mutagenesis, and X-ray crystallography. The crystal structures of *Rg*GH98 in complex with BgA trisaccharide (BgAtri) and of *Rg*GH98 E411A with BgA II revealed a dedicated hydrogen network of residues, which were shown by site-directed mutagenesis to be critical to the recognition of the BgA epitope. We demonstrated experimentally that *Rg*GH98 is part of an operon of 10 genes that is overexpresssed in vitro when *R*. *gnavus* ATCC 29149 is grown on mucin as sole carbon source as shown by RNAseq analysis and RT-qPCR confirmed *Rg*GH98 expression on BgA II growth. Using MALDI-ToF MS, we showed that *Rg*GH98 releases BgAtri from mucin and that pretreatment of mucin with *Rg*GH98 confered *R*. *gnavus* E1 the ability to grow, by enabling the E1 strain to metabolise BgAtri and access the underlying mucin glycan chain. These data further support that the GH repertoire of *R*. *gnavus* strains enable them to colonise different nutritional niches in the human gut and has potential applications in diagnostic and therapeutics against infection.

## Abstract 2

Ruminococcus gnavus is a human gut symbiont that displays strain-specific repertoires of glycoside hydrolases (GH). This study finds that the R. gnavus enzyme GH98 is specific for blood group A (BgA) tetrasaccharide type II, and that it can release BgA trisaccharide from mucins, showing that the GH repertoire of R. gnavus strains enables them to colonise different nutritional niches in the human gut.

## Introduction

The gut microbiota plays a major role in human health and an alteration in its structure and function has been implicated in several diseases (for a review, see [1]). In the colon, mucus covering the epithelium is critical to maintain a homeostatic relationship with the gut microbiota by harbouring a microbial community at safe distance from the epithelium surface [2]. The mucin glycans composing the mucus layer provide binding sites and a sustainable source of nutrients to the bacteria inhabiting the mucus niche [3–5]. Mucins are large glycoproteins with a high carbohydrate content of up to 80%. Mucin-type *O*-glycans consist of *N*-acetylgalactosamine (GalNAc), Gal and *N*-acetylglucosamine (GlcNAc) containing glycan chains usually capped by fucose (Fuc) and/or sialic acid, giving rise to blood groups A, B, and H and sialyl-Lewis epitopes [6–9]. The peripheral terminal epitopes show considerable variation with a decreasing gradient of Fuc and ABH blood group expression and an increasing gradient of sialic acid from the ileum to the colon [7]. For example, blood group H and A antigenic determinants were shown to be present exclusively in the ileum and cecum, whereas blood group Sd(a)/Cad-related epitopes were found to increase along the length of the colon [6,7]. These gradients are reversed in mice, where the small intestine is dominated by sialylated structures and the colon with those terminating in Fuc [10].

Access to these glycan chains require a complement of glycoside hydrolases (GHs) produced by bacteria across the phyla constituting the human gut microbiota [4]. A small number of microbial GH families have ben shown to be active on blood group antigens [11–21]. Among these, the GH109 family includes exoglycosidases that remove the nonreducing terminal A or B antigen-determining GalNAc or Gal residues, respectively, converting the antigen to the H-type (O-type) [11], whereas the GH98 family includes endo-β-1,4-galactosidases acting on the galactosyl-β-1,4-N-acetylglucosamine linkage found in type 2 carbohydrate blood group antigens containing (Fucα1–2)Galβ1-4GlcNAc. The GH98 enzymes characterised to date are E-ABase from *Clostridium perfringens* ATCC 10543 and *Sp*3GH98 from *Streptococcus pneumoniae* SP3-BS71 which are capable of liberating GalNAcα1-3(Fucα1–2)Gal and Galα1-3(Fucα1–2)Gal trisaccharides from glycoconjugates containing blood group A and B glycotopes, respectively, whereas *Sp*4GH98 from *Streptococcus pneumoniae* TIGR4 displays specificity for Fucα1-2Gal of the Lewis Y antigen [14,16].

*Ruminococcus gnavus* is a prevalent member of the gut microbial community belonging to the Firmicutes division [22,23]. *R*. *gnavus* is an early coloniser of the human gut [24] but persists in healthy adults as one of the 57 species detected in more than 90% of human faecal samples by metagenomic sequencing [22]. Interestingly, an increasing number of studies are reporting a disproportionate representation *R*. *gnavus* in diseases such as inflammatory bowel disease [25]. In our previous work, we showed that the mucin-foraging strategy of *R*. *gnavus* is strain specific [26] and associated with the expression of specific GHs active against terminal epitopes, including GH33 intramolecular *trans*-sialidase (IT-sialidase) [27–29] and GH29 or GH95 fucosidases [30]. Interestingly, a gene encoding for a predicted GH98 blood-group endo-β-1,4-galactosidase was found to be exclusively present and induced in *R*. *gnavus* strains grown on mucins [26,28]. Since *R*. *gnavus* ATCC 29149 but not E1 contained a GH98 encoding gene and was able to grow on mucin as sole carbon source, we hypothesised that GH98 could be a critical molecular determinant in confering mucin glycan utilisation capacity to *R*. *gnavus* strains. In order to test this hypothesis, we determined the expression, genetic organisation, and substrate specificity of *R*. *gnavus* ATTC 29149 GH98 enzyme (*Rg*GH98) and showed that mucin treatment with *Rg*GH98 confered *R*. *gnavus* E1 strain the ability to grow on mucins by enabling E1 to metabolise BgA and access the underlying mucin glycan chain. These further data support the role of GHs in the adaptation of *R*. *gnavus* strains to distinct nutrional niches.

## Results

### *R*. *gnavus* GH98 displays substrate specificity for blood group A antigen and mucin

**Fig 1:**
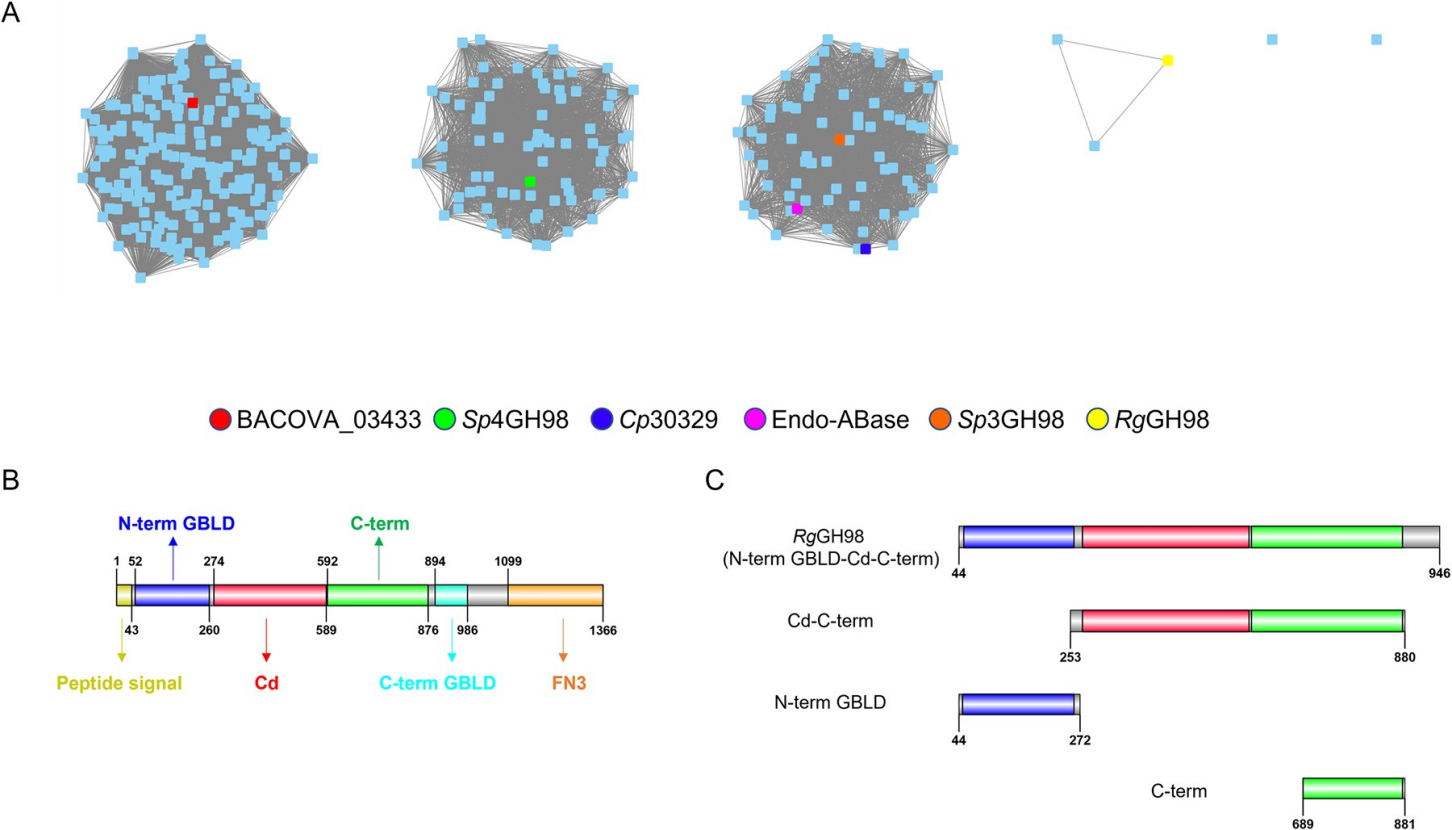
In silico analysis of *Rg*GH98. (**A**) SSN analysis of GH98 family. Amino acid sequences were from the CAZy database (www.cazy.org). A total of 355 GH98 sequences were analysed by SSN with an alignment score of 120. Each node (blue dot) represents one protein sequence. The functionally characterised enzymes are colour coded. (**B**) Domain organisation of *Rg*GH98 encompassing the N-term GBLD (52–260 aa in blue), the Cd (274–589 aa in red), the C-term domain (592–876 aa in green), the C-term GBLD (894–986 aa in turquoise), and the C-term FN3 domain (1,099–1,366 aa in amber). (**C**). Schematic representation of *Rg*GH98 constructs. Recombinant *Rg*GH98 (44–946 aa) encompassing the N-term GBLD (blue), Cd (red), and C-term (green) domains; the individual N-term GBLD domain (blue); Cd-C-term domain (red-green); and C-term domain (green). Graphs were made using Cytoscape v3.4.0 (**A**) and IBSv1.0 (ref = https://doi.org/10.1093/bioinformatics/btv362) (**B** and **C**). aa, amino acid; Cd, central/catalytic domain; C-term, C-terminal; C-term FN3, C-terminal fibronectin type 3; C-term GBLD, C-terminal galactose-binding-like domain; N-term GBLD, N-terminal galactose-binding-like domain; SSN, sequence similarity network.

Sequence similarity network (SSN) analysis of GH98 family members revealed 3 major clusters with functionally characterised GH98 enzymes from the CAZy database (www.cazy.org). The GH98 sequence from *R*. *gnavus* ATCC 29149 was found outside the 3 main clusters (**Fig 1A**), which may suggest differences in substrate specificity. *R*. *gnavus* ATCC 29149 putative GH98, *Rg*GH98, is predicted to be a modular protein of 1,366 amino acids (aa) including a predicted N-terminal 43 aa peptide signal typical of gram-positive bacteria. *Rg*GH98 modular structure consists of an N-terminal galactose-binding-like domain (N-term GBLD) (52–260 aa), a central/catalytic domain (Cd) covering 274–589 aa, a C-terminal (C-term) domain (592–876 aa), a C-term GBLD (894–986 aa), and a C-term fibronectin type 3 domain (1,099–1,366 aa) (**Fig 1B**).

Sequence alignments were carried out with functionally characterised GH98 enzymes, namely the GH98 endogalactosidase E-ABase from *C*. *perfringens* [16] and the GH98 enzymes from *S*. *pneumoniae* TIGR4 (*Sp*4GH98) and SP3-BS71 (*Sp*3GH98) [14]. *Rg*GH98 Cd shows 36% amino acid identity with the catalytic domains of *C*. *perfringens* E-ABase, 34% with *Sp*3GH98, and 30% with *Sp*4GH98. *Rg*GH98 C-term shares 31% amino acid identity with the C-term domains of *C*. *perfringens* E-ABase, 30% with *Sp*3GH98, and 26% with *Sp*4GH98. The sequence similarity between C-term domains across GH98 enzymes [31] may also reflect its close spatial interaction with the catalytic domain, as reported for *Sp*4GH98 [14]. The sequences of N-term GBLD and C-term GBLD share 26% identity. Additionally, *Rg*GH98 N-term GBLD is 23% identical to CBM51-1 of *Sp*3GH98, while the C-term GBLD is about 25% identical to the CBM47-1 of *Sp*4GH98. Guided by amino acid sequence-based comparison, we cloned the RGna_RS10325 gene encompassing the N-term GBLD, Cd, and C-term domains (which we referred to as *Rg*GH98 in the rest of the study) as well as the N-term GBLD, C-term, and Cd-C-term domains individually (**Fig 1C**). The signal peptide (1–43 aa), the C-term GBLD (894–986 aa), and C-terminal fibronectin type 3 domain (1,099–1,366 aa) were not included in any of the expression constructs. *Escherichia coli* Tuner DE3 pLacIs strain was chosen as heterologous host as it does not display any endogenous β-galactosidase activity (due to the deletion of the LacZ gene) that may interfere with the enzymatic characterisation of the recombinant enzymes.

**Fig 2:**
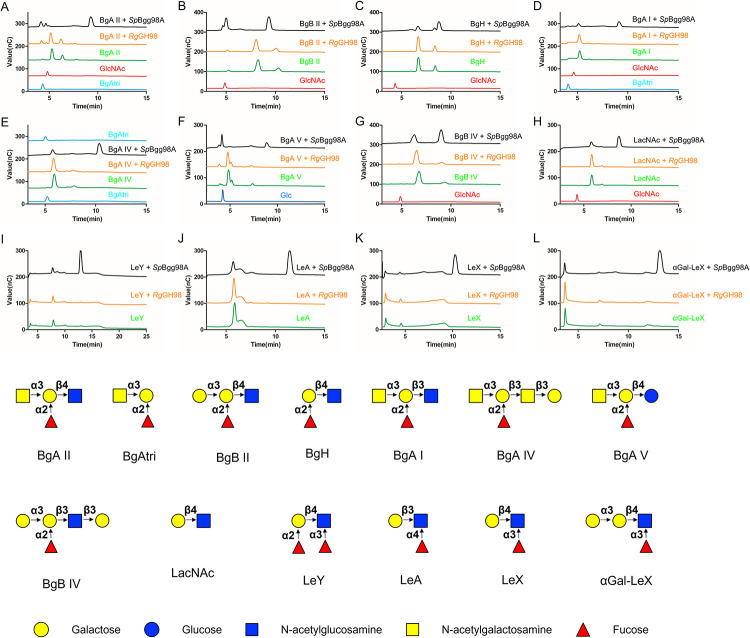
HPAEC-PAD analysis of *Rg*GH98 against complex oligosaccharides. BgA II (**A**), BgB II (**B**), BgH (**C**), BgA I (**D**), BgA IV (**E**), BgA V (**F**), BgB IV (**G**), LacNAc (**H**), LeY (**I**), LeA (**J**), LeX (**K**), αGal-LeX (**L**). The oligosaccharide structures are provided in the lower panel. Monosaccharide symbols follow the Symbol Nomenclature for Glycans system [33]. αGal-LeX, α1,3Gal-Lewis X; BgA I, blood group A tetrasaccharide type I; BgA II, blood group A tetrasaccharide type II; BgA IV, blood group A pentasaccharide type IV; BgA V, blood group A tetrasaccharide type V; BgB II, blood group B tetrasaccharide type II; BgB IV, blood group B pentasaccharide type IV; BgH, blood group H trisaccharide; HPAEC-PAD, high-pH anion exchange chromatography with pulsed amperometric detection; LacNAc, *N*-Acetyllactosamine; LeA, Lewis A trisaccharide; LeX, Lewis X trisaccharide; LeY, Lewis Y tetrasaccharide.

The recombinant *Rg*GH98 enzyme showed no activity against the synthetic substrate GlcNAc-*p*NP. The activity of *Rg*GH98 was then tested against tetra and penta blood group antigens including BgA I (GalNAcα1–3[Fucα1–2]Galβ1-3GlcNAc), BgA II (GalNAcα1–3[Fucα1–2]Galβ1-4GlcNAc), BgA IV (GalNAcα1–3[Fucα1–2]Galβ1-3GalNAcβ1-3Gal), BgA V (GalNAcα1–3[Fucα1–2]Galβ1-4Glc), BgB II (Galα1–3[Fucα1–2]Galβ1-4GlcNAc), BgB IV (Galα1-3(Fucα1–2)Galβ1-3GalNAcβ1-3Gal), BgH (Fucα1-2Galβ1-4GlcNAc), as well as LeY (Fucα1-2Galβ1–4[Fucα1–3]GlcNAc), LeA (Galβl-3[Fucα1–4]GlcNAc), LeX (Galβ1–4[Fucα1–3]GlcNAc), and LacNAc (Galβ1-4GlcNAc) (see structures in **Fig 2**). *Sp*Bgg98A from *S*. *pneumoniae* was used as control and the products of the reactions analysed by HPAEC-PAD (**Fig 2).** Among all the oligosaccharides tested, BgA II tetrasccharide was the sole substrate hydrolysed by *Rg*GH98. The chromatograms clearly showed the appearance of peaks corresponding to GlcNAc and BgAtri (GalNAcα1–3(Fucα1–2)Gal) and a decrease in the peak corresponding to BgA II (**Fig 2A**), in line with the cleavage of Galβ1-4GlcNAc glycosidic bond. In contrast, no reaction product was detected when other types of blood group A, blood group antigen B or H, LacNAc or lewis antigens were used as substrates (**Fig 2B–2L**). *Rg*GH98 was further tested on a range of Fuc-containing oligosaccharides including 2′FL (Fucα1,2Galβ1,4Glc), 3FL (Galβ1–4[Fucα1–3]Glc), DFL (Fucα1-2Galβ1-4(Fucα1–3)Glc), Fucα1-6GlcNAc, and α-1,6-fucosylated biantennary *N*-glycan (FA2G2) as well as on xyloglucan and arabinoxylan, which were shown be susbtrates of GH98 enzymes [32], but no activity was detected by HPAEC-PAD for any of these compounds (**S1 Fig**).

**Fig 3:**
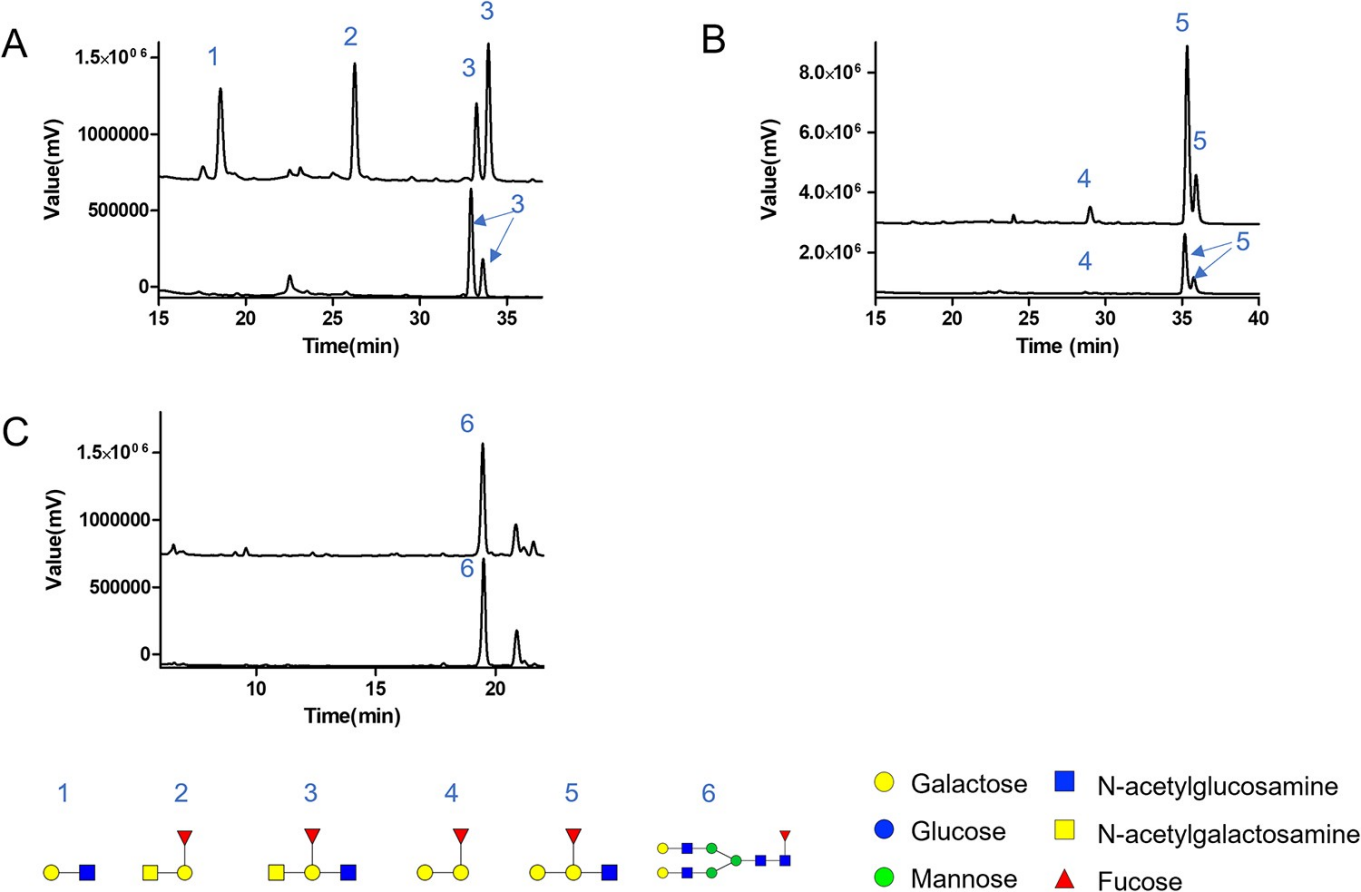
LC-FD-MS/MS analysis of *Rg*GH98 against complex oligosaccharides. Chromatograms of the enzymatic reaction between *Rg*GH98 and BgA II (**A**), BgB II (**B**), and FA2G2 (**C**). The lower chromatogram corresponds to the reaction with no enzyme (substrate only), while the upper lane corresponds to the reaction with *Rg*GH98. Monosaccharide symbols follow the Symbol Nomenclature for Glycans system [33]. BgA II, blood group A tetrasaccharide type II; BgB II, blood group B tetrasaccharide type II; LC-FD-MS/MS, liquid chromatography with fluorescence detection and mass spectrometric detection.

*Rg*GH98 susbstrate specificity was further confirmed by LC-FD-MS/MS analysis indicating that *Rg*GH98 liberates the terminal BgAtri of the A antigen while no reaction products were detected when BgB II or FA2G2 were used as substrate (**Fig 3**). Kinetics and optimum pH analyses were then performed using BgA II as a substrate. The pH optimum was found to be pH 5 (**S2A Fig).** The kinetic parameters were determined at the optimum pH by calculating the initial rate of reaction with increasing BgA II concentrations (**S2B Fig**). The recombinant enzyme showed a *k*_cat_ of 0.17 min^−1^ and a *K*_M_ of 516.9 μM against this substrate (**Table 1**).

**Table 1:** Kinetic parameters of *Rg*GH98 on BgA II.

Enzyme	Substrate	Vmax (μM·min^−1^)	*K*_M_ (μM)	*k*_cat_ (min^−1^)	Kcat/Km (μM^−1^min^−1^)
*Rg*GH98	BgA II	1.702 ± 0.08	516.9 ± 71	0.17 ± 0.008	3.3 10^−4^ ± 4.8 10^−5^

**Fig 4:**
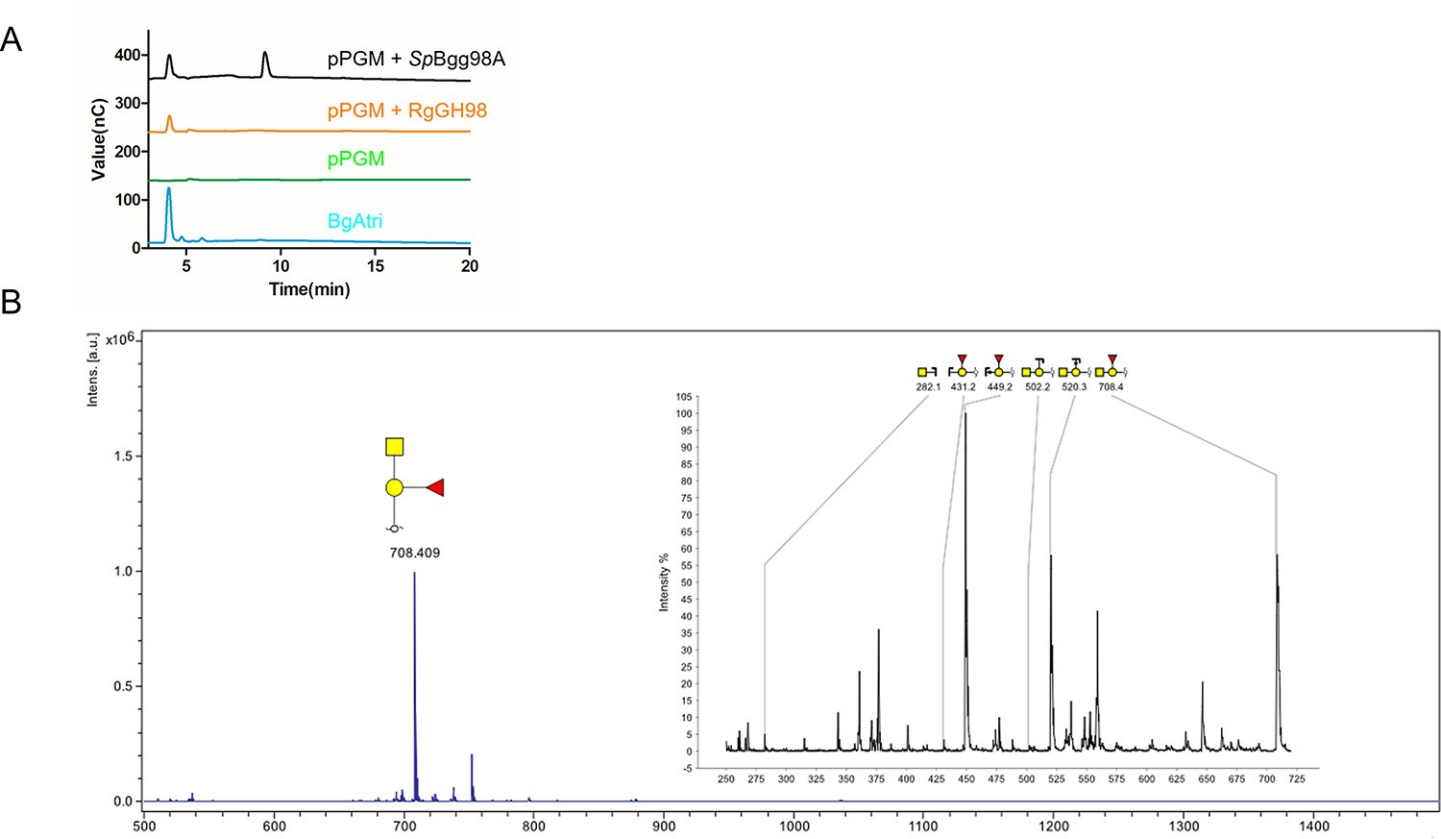
*Rg*GH98 enzymatic activity against mucin. (**A**) HPAEC-PAD analysis of *Rg*GH98 against pPGM. (**B**) MS analysis of the released glycan after treatment of pPGM with *Rg*GH98. Inlay: fragmentation of the glycan peak at 708 Da. The results show that Fuc is bound onto the galactose at the reducing end, supporting the identification of this glycan as BgAtri. Monosaccharide symbols follow the Symbol Nomenclature for Glycans system [33]. BgAtri, BgA trisaccharide; Fuc, fucose; HPAEC-PAD, high-pH anion exchange chromatography with pulsed amperometric detection; MS, mass spectrometry; pPGM, purified pig gastric mucin.

*Rg*GH98 also showed activity against purified pig gastric mucin (pPGM), with HPAEC analysis showing a peak corresponding to BgAtri as also observed using *Sp*Bgg98A as a control (**Fig 4A**). Confirmation of the BgAtri structure was obtained by MALDI-ToF MS analysis following dialysis of the enzymatic reaction, and reduction and permethylation of the dialysate. The MS spectrum showed a dominant peak at 708 Da, corresponding to a permethylated, sodiated trisaccharide composed of a deoxy-hexose, a hexose, and an *N*-acetyl-hexosamine. Fragmentation of this species showed that the deoxy-hexose was linked to the hexose at the reducing end, as determined by the characteristic fragments at 431 and 449 Da (**Fig 4B**). Together, the HPAEC-PAD and MS/MS analyses confirmed that BgAtri was released from mucin following *Rg*GH98 hydrolysis of the Galβ1-4GlcNAc glycosidic linkage in BgA II.

### Structural basis of *R*. *gnavus* GH98 substrate specificity

**Fig 5:**
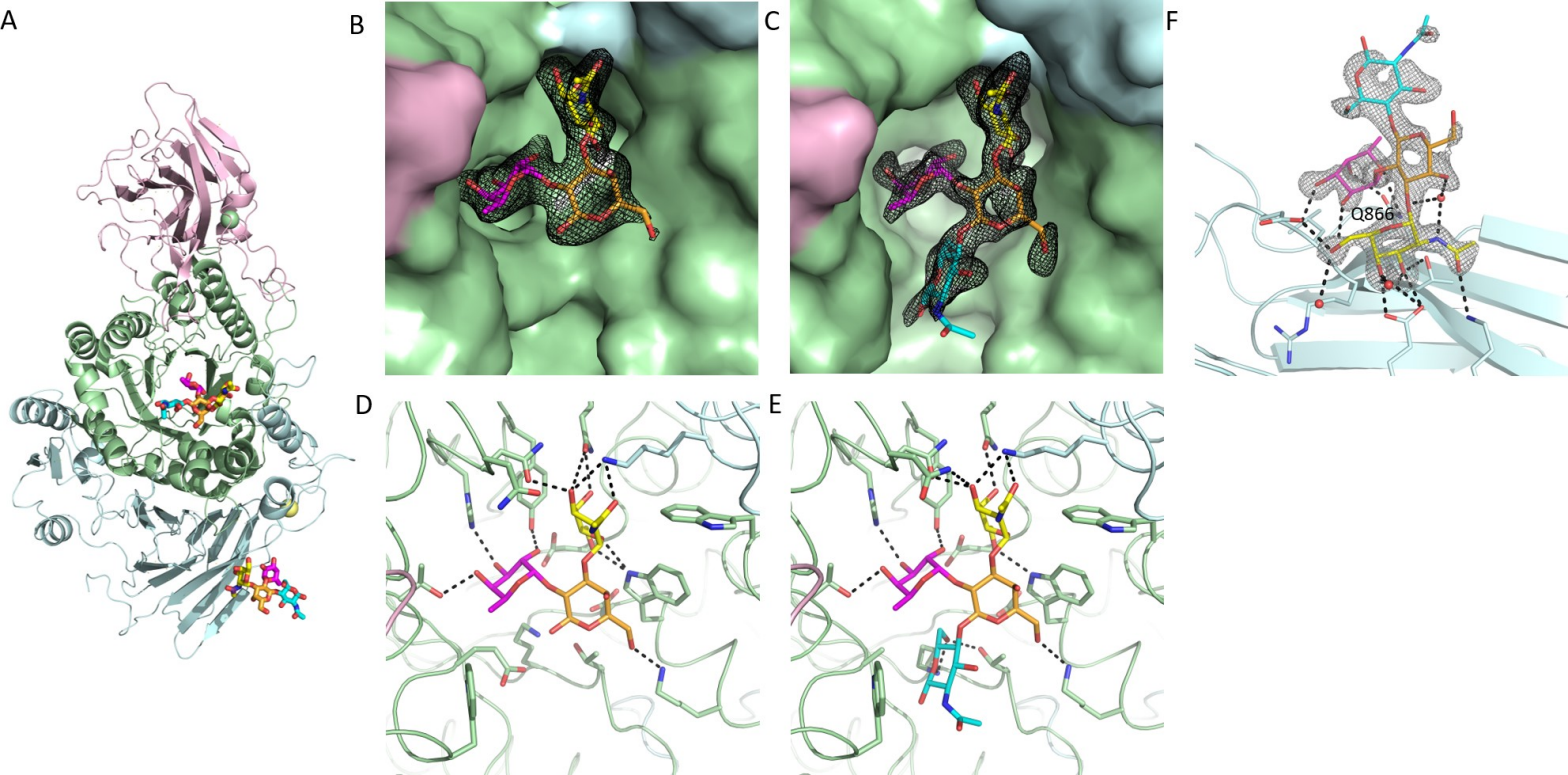
Crystal structures of *Rg*GH98. (**A**) Cartoon representation of *Rg*GH98. N-term GBLD in pink, Cd in green, and C-term domains in light cyan. BgA II molecules are shown in stick representation. Fuc is coloured pink, Gal in orange, GlcNAc in cyan, and GalNAc in yellow. The yellow and green spheres represent bound calcium and magnesium ions, respectively. Fo-Fc omit map electron density for **(B)** BgAtri and **(C)** BgA II bound in the active site (σ level of 3). (**D**) Active site with BgAtri bound and nearby residues highlighted. Putative hydrogen bonding interactions are shown with black dashed lines. (**E**) *Rg*GH98 E411A with BgA II bound. **(F)** BgA II bound to the C-term domain. Fo-Fc omit map electron density for the bound carbohydrate is shown with a σ level of 3. BgA II, blood group A tetrasaccharide type II; BgAtri, BgA trisaccharide; Cd, central/catalytic domain; C-term, C-terminal; Fuc, fucose; GalNAc, N-acetylgalactosamine; GlcNAc, N-acetylglucosamine; N-term GBLD, N-terminal galactose-binding-like domain.

*Rg*GH98 was first crystallised as the absence of ligand showing electron density for residues 49 to 893 (see **Table 2** for data collection and refinement statistics). As expected from the construct, *Rg*GH98 adopts a modular structure with 3 domains. The N-term domain identified as GBLD, residues 55 to 260, presents a β-sandwich fold (**Fig 5A**). Two loops, comprising residues 79 to 105 and 138 to 163, extend from the GBLD and wrap around the Cd. Cd, residues 274 to 589, adopts an (α/β)_8_ barrel fold with similarity to *Sp*3GH98 (1.14 rmsd, pdb 2WMI, 4D71) and *Sp*4GH98 (1.31 rmsd, pdb 2WMG) from *S*. *pneumoniae* [14,34]. Structural alignment to homologous GH98 enzymes identified the binding pocket and the general acid catalytic residue as Glu 411 (see **S3 Fig),** which is present in a cleft in the centre of the Cd. The C-term domain, residues 592 to 876 aa, with a central β-sandwich module follows.

**Table 2:** *Rg*GH98 crystal structures: Data collection, statistics, and refinement.

	*Rg*GH98	*Rg*GH98-BgAtri	*Rg*GH98 E411A-BgA II
Data collection			
Space group	P21	P21	P212121
Cell dimensions			
*a*, *b*, *c* (Å)	104.94, 85.57, 112.62	107.60, 86.95, 110.20	78.26, 103.66, 113.28
*α*, *β*, *γ* (°)	90, 99.10, 90	90, 100.33, 90	90, 90, 90
Resolution	103.9–2.20 (2.23–2.20)	108.41–1.95 (1.98–1.95)	76.47–1.65 (1.68–1.65)
Rmeas	0.149 (0.717)	0.172 (1.748)	0.196 (3.608)
*I/σI*	5.8 (1.7)	5.8 (1.1)	6.6 (0.7)
Completeness (%)	99.5 (92.2)	99.9 (97.7)	100 (99.1)
Redundancy	3.5 (3.5)	5.4 (5.4)	8.8 (8.0)
Total reflections	353,294 (16,100)	788,430 (37,745)	980,070 (43,087)
Unique reflections	100,422 (4,614)	145,680 (37,745)	111,311 (5,397)
Refinement			
Resolution	103.9–2.20	108.41–1.95	76.47–1.65
No. reflections	100,402	145,485	111,181
R_work_/R_free_ (%)	0.183/0.209	0.178/0.215	0.171/0.200
No. atoms			
Protein	13,458	13,360	6,700
Ion	6	8	2
Carbohydrate		72	100
Water	1,127	834	708
B-factors (Å^2^)			
Protein	16.7	18.4	25.8
Ion	40.2	43.8	21.3
Carbohydrate		24.0	46.9
Water	42.5	42.9	32.1
r.m.s deviations			
Bond lengths (Å)	0.01	0.007	0.04
Bond angles (°)	1.4	1.4	1.20

Incubation of *Rg*GH98 crystals with BgAtri produced a complex with clear electron density for the BgAtri in the binding pocket near Glu 411 (**Fig 5B and 5D, S4A Fig)**. *Rg*GH98 makes hydrogen bond interactions with each of the 3 sugar residues present in BgAtri (GalNAcα1-3(Fucα1–2)Gal-): Tyr 289, His 330, and Thr 371 with Fuc; Lys513 with Gal; and Gln 305, Asn 332, and Lys 788 with GalNAc, respectively. No large movements in side chain positions were observed upon ligand binding (**S4A Fig**). The difference between BgA and BgB is the presence of terminal GalNAc and Gal at the nonreducing end of A and B antigens, respectively. GalNAc has an N-acetyl moiety (-NHCOCH_3_) at the C2 position whereas Gal has a smaller–OH at this position. Lys 788, present on an extension from the C-term domain, forms part of a hydrogen bonding network with the GalNAc *N*-acetyl moiety and a ring hydroxyl, forming a stable structure (**Fig 5D and 5E**). This residue is conserved in *Sp3*GH98 (as Lys927) (**S4B Fig**), a GH that does not demonstrate preference of BgA>BgB. However, unique to *Rg*GH98, Gln 305 and Trp 528 provide additional interactions, bringing the terminal GalNAc into a location conducive to hydrogen bond with Lys 788. In concert, these residues are proposed, to provide the observed BgA>BgB specificity. Gln 305, Trp 528, and the GalNAc binding site are unique to *Rg*GH98 as compared to *Sp*3GH98 (**S4B Fig**), *Sp*4GH98 (**S4C Fig**), *Ea*bC, *Cp*e0329 [16], and BACOVA_03433 [32]. Gln 305 corresponds to a Trp residue in these GH98 enzymes and Trp528 is either Asp or Asn apart for *Sp*4GH98, where it is a Met.

We generated an *Rg*GH98 E411A mutant and obtained a complex with the unprocessed carbohydrate following incubation of *Rg*GH98 E411A crystals with BgA II (GalNAcα1–3[Fucα1–2]Galβ1-4GlcNAc) (**Fig 5C and 5E)**. When the complexes with BgAtri (trisaccharide) and BgA II (tetrasaccharide) were overlaid, the GlcNAc residue was found to be very close to Glu 411 at <2 Å in the tetrasaccharide complex (**S4D Fig**). The GlcNAc spatial arrangement is in contrast to the *Sp*3GH98 complex, in which the GlcNAc residue is pivoted with respect to Gal (**S4E Fig**). It is proposed that Glu 411 adopts a different rotamer in the presence of substrate. Changes in conformation of catalytic glutamate side chains in GHs have been observed using neutron crystallography, alternating between upward and downward conformations with the protonated form being in the downward orientation [35]. As well as bringing the side chain into a position from which a steric clash is avoided, this shift also provides a change in local environment, which may alter residue’s pK_a_, promoting catalysis.

Density for an additional BgA II tetrasaccharide was observed at the C-term domain (**Fig 5F)**. Interactions are primarily with the terminal GalNAc of BgA II, specifically with Lys713, Glu734, Glu814, Arg816, Thr817. Additionaly, hydrogen bonding interactions are formed with the Fuc residue by Glu814, Leu815, and Gln866. It is proposed that this identifies the N-term domain as a carbohydrate binding domain of *Rg*GH98. Definitive data identifying a definitive binding partner for the N-term proposed GBLD has not yet been captured.

In the *Rg*GH98 E411A-BgA II complex, 2 metal ions were modelled (**Fig 5A, S4F and S4G Fig**). The first, a magnesium ion, in the GBLD N-term, coordinated by the side chains of Asp 79, Tyr 84, and Glu 255 and the backbone carbonyls of Leu 76 and Met 254. The second, a calcium ion, in the C-term domain, coordinated by Asp 812, Asn 813, and Gln 846, with additional water models filling the octahedral geometry. Additional calcium and magnesium ions were modelled in the *Rg*GH98–BgAtri complex and *Rg*GH98 apo crystal structures. However, in these experiments both ions were present at a concentration of 50 mM, increasing the likelihood of nonspecific binding. To confirm the presence of metals in solution, *Rg*GH98 was analysed by inductively coupled plasma mass spectrometry (ICP-MS). The highest amount of metal ion detected in the protein was calcium (Ca^2+^ = 115.3 μmoles), followed by Zinc (Zn^2+^ = 21.88 μmoles) and magnesium (Ca^2+^ = 19.57 μmoles) **(S1 Table**). The ratio of calcium to *Rg*GH98 was estimated to be approximately 1.5:1.

**Fig 6:**
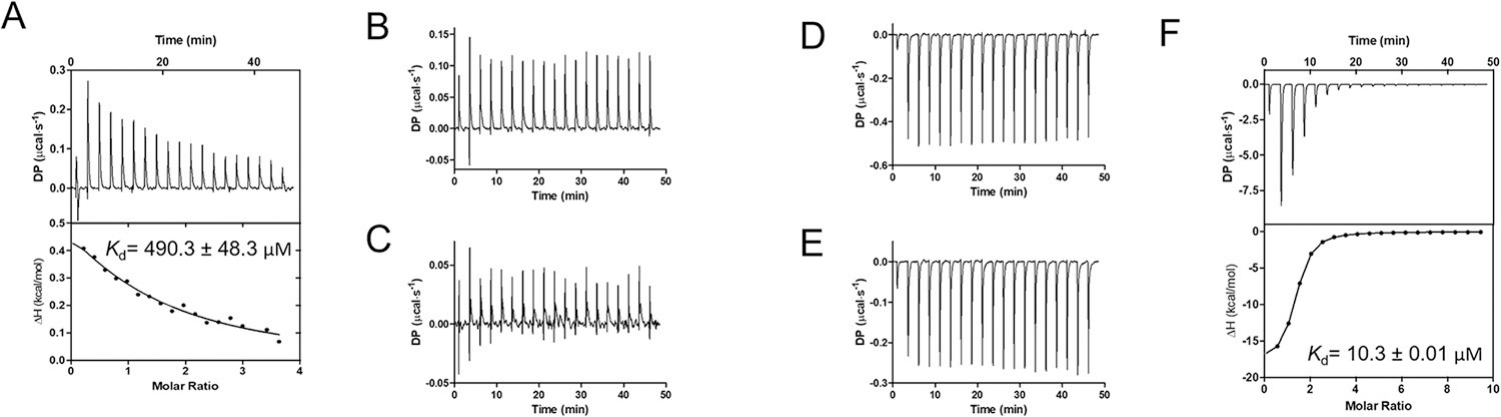
ITC analysis of proteins binding to blood group antigens. ITC isotherms of *Rg*GH98 E411A against BgA II (**A**), BgB II (**B**), BgH (**C**). ITC isotherms of UEA I lectin against BgA II (**D**), BgB II (**E**), BgH (**F**). BgA II, blood group A tetrasaccharide type II; BgB II, blood group B tetrasaccharide type II; BgH, blood group H trisaccharide; DP, differential power; ITC, isothermal titration calorimetry.

Based on the crystal structures of *Rg*GH98 in complex with BgA oligosaccharides and sequence alignments with structurally characterised GH98 enzymes (**S3 Fig**), *Rg*GH98 site-directed mutants, K788A, W528A, W528D, Q305A, and Q305W, were produced (**S5A Fig**) and tested against BgA II. K788A, W528A, W528D, and Q305W lost the enzymatic activity towards this substrate, whereas Q305A remained active (39.70%) (**S5B Fig**). To investigate the potential impact of the mutations on *Rg*GH98 stability, the recombinant enzymes were subjected to differential scanning fluorimetry (DSF) analysis. K788A showed similar melting temperature (Tm) values as *Rg*GH98 while the Tm values of the other mutants were relatively lower (**S5C Fig**), indicating that, for K788A, the loss of activity may be attributed to the interaction of Lys 788 with BgA II. We next used isothermal titration calorimetry (ITC) to determine the binding kinetic parameters of *Rg*GH98 E411A mutant towards blood group A, B, and H antigens (**Fig 6, S2 Table**). The UEA I lectin with affinity to ABO blood group was used as a control. *Rg*GH98 E411A bound to BgA II with a *K*_d_ of 490.3 μM (**Fig 6A, S2 Table**). No binding was observed between *Rg*GH98 E411A and BgB II or BgH whereas UEA I bound to BgH with a *K*_d_ of 10.3 μM (**Fig 6B, 6C and 6D**). The value of *K*_d_ is in the same range as *K*_M_ (516.9 μM), indicating k_−1_ >> *k*_cat_ (*K_M_* = (*k*_−1_+*k_cat_*)/*k*_1_, *K_d_* = *k*_−1_/*k*_1_), i.e., dissociation is much faster than catalysis, in line with *Rg*GH98 low *k*_cat_.

**Fig 7:**
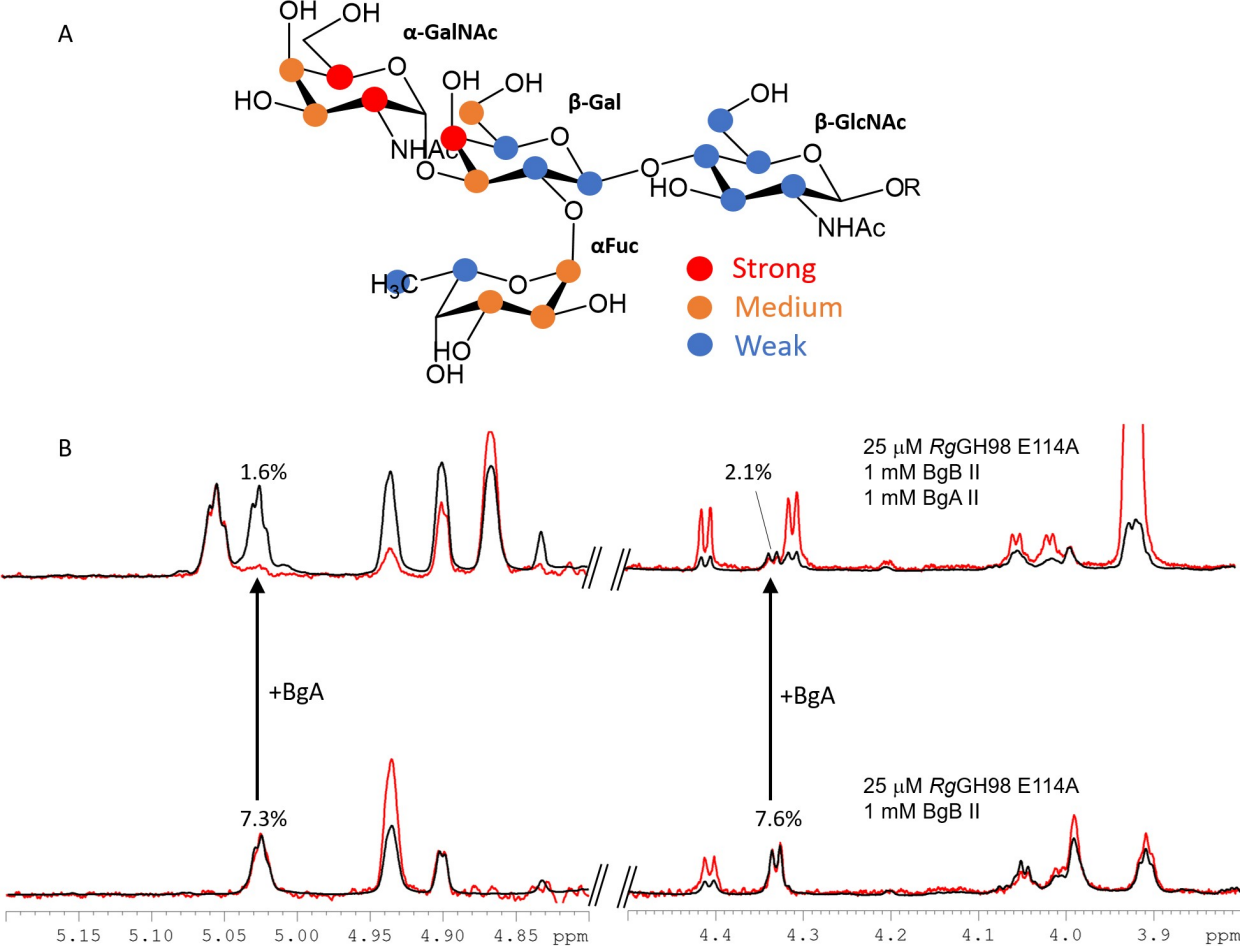
STD NMR spectroscopy of the interactions of blood group antigens with *Rg*GH98. (**A**) STD NMR binding epitope mapping of BgA II upon its interaction with *Rg*GH98 EA411A, based on normalised saturation transfer intensities (0%–100%) from initial slopes of the corresponding STD NMR build-up curves; selective protein irradiation at 0.0 ppm. Colour indicates weak (blue), medium (yellow) and strong (red) intensities. Large normalised STD intensities indicate closer ligand contacts with the surface of the protein in the bound state. R ≡ − CH2CH2N3. Initial slopes and normalised STD values are reported in S5 Table. (**B**) Zoomed regions of STD NMR competition experiments for binding of BgB II to *Rg*GH98 EA411A. The STD NMR intensities of BgB II were highly reduced when adding BgA II in equimolar concentration to BgB II (1 mM). The selected regions highlight the effect for the 2 best resolved signals of BgB II in the final mixture. An average reduction of ca. 75% in BgB II intensities demonstrated a much higher affinity of BgA II towards the protein, demonstrating the preference of *Rg*GH98 for this ligand. BgA II, blood group A tetrasaccharide type II; BgB II, blood group B tetrasaccharide type II; STD NMR, saturation transfer difference nuclear magnetic resonance spectroscopy.

In order to gain further structural insights into *Rg*GH98 unique ligand specificity to blood group A, saturation transfer difference nuclear magnetic resonance spectroscopy (STD NMR) studies [36] were conducted with *Rg*GH98 E411A mutant in the presence of BgA II, BgB II (**Fig 7**), and BgH (**S6 Fig**). Transfer of magnetization as saturation from the protein to the ligand was observed for BgA II (**Fig 7**). The main contact was at the GalNAc terminal ring, in agreement with *Rg*GH98 activity on this substrate and with the crystal structures of the complexes of *Rg*GH98 and *Rg*GH98 E411A with BgAtri and BgA II, respectively. In both structures, the terminal GalNAc makes the largest number of contacts with the protein, followed by the Fuc ring, in excellent agreement with the experimental mapping of the binding epitope of BgA II determined by NMR (**Fig 7A**), confirming the observation of specific binding under the STD NMR experimental conditions. Furthermore, no binding could be detected to the recombinant individual N-term or C-term domains (**S6 Fig, left panel**). STD NMR also showed binding of *Rg*GH98 to BgB II but with highly reduced affinity in comparison to BgA II as the binding was almost abolished when BgA II was added to the sample (**Fig 7B**). No binding to BgH was detected by STD NMR (**S6 Fig, right panel**). Addition of BgA II to the BgH/*Rg*GH98 E411A reaction led to strong STD NMR signals characteristic of the binding to BgA, supporting the specificity of the recognition (**S6 Fig, right panel**).

To explore the full ligand specificity of *Rg*GH98, E411A and individual C-term and N-term GBLD were screened against 585 glycans from the Core H glycan microarray at the Consortium for Functional Glycomics (CFG) (**S7 Fig**). The proteins bound with low intensity to a range of ligands with a preference for glycan ID389, which has the epitope of αGal-LeA (Gal-α1,3-Gal-β-1,3(Fuc1,4)GlcNAc) (ID = 389) (**S7 Fig**). However, the low relative fluorescence units (RFUs) (<150) obtained against these sugars do not allow us to infer conclusive results regarding glycan-binding specificity.

### *R*. *gnavus* ATCC 29149 GH98 is part of an operon dedicated to mucin and blood group A utilisation

**Fig 8:**
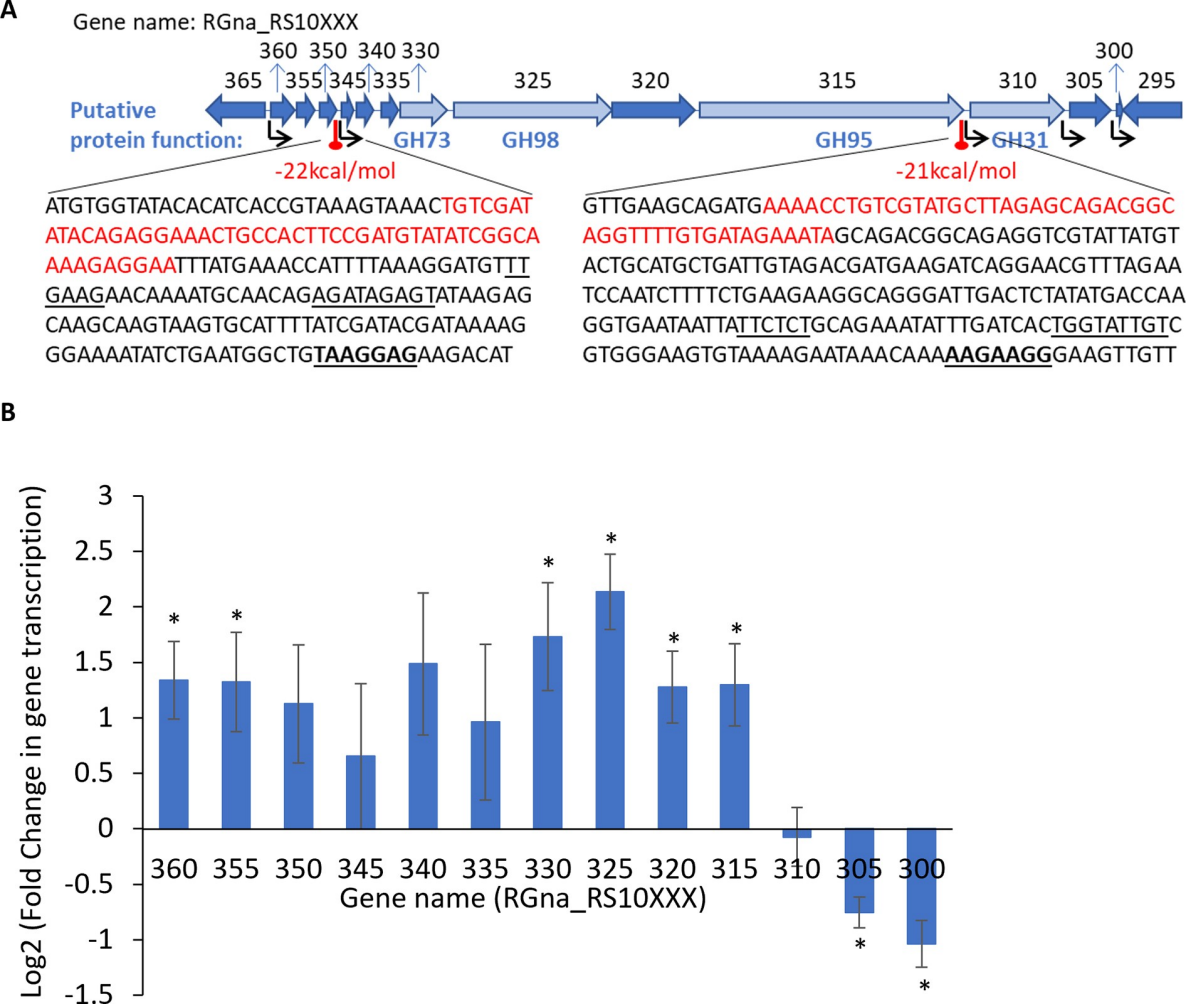
Analysis of *R*. *gnavus* ATCC 29149 GH98 cluster. (**A**) In silico analysis of the predicted GH98 operonic structure. Thirteen genes (RGna_RS10360 to RGna_RS10300) are located on the same DNA strand. Red circles above thick vertical lines indicate potential stem-loop structures that might act as Rho-independent transcriptional terminators. The free energy of the thermodynamic ensembles (in kcal/mol) is marked in red. Black arrows show predicted promoters. The insets show the DNA sequences between genes RGna_RS10350 and RGna_RS10345 and between genes RGna_RS10315 and RGna_RS10310; sequences of the potential transcriptional terminators are shown in red, while sequences of the predicted −35 and −10 regions of the promoter are underlined. Potential RBS sequences are in bold and underlined. (**B**). RNAseq Log2 of the fold change in gene transcription for RGna_RS10360 to RGna_RS10300, when *R*. *gnavus* ATCC 29149 was grown with pPGM as compared to Glc as sole carbon source. It was calculated using averages of 4 biological replicates per growth condition. *Adjusted *p*-value <0.05. Underlying data can be found in S1 Data. Glc, glucose; pPGM, purified pig gastric mucin; RBS, ribosome binding site.

Analysis of *R*. *gnavus* ATCC 29149 genome revealed that the gene encoding *Rg*GH98 (RGna_RS10325) is part of a 21.6-kb cluster containing a total of 13 genes on the same DNA strand (RGna_RS10300 to RGna_RS10360), 7 upstream and 5 downstream of the GH98 gene. As shown in **Fig 8A**, 3 GH-encoding genes (RGna_RS10330, RGna_RS10315, and RGna_RS10310 coding for GH73, GH95, and GH31, respectively) were identified in this cluster. In silico analysis of the intergenic regions identifed transcriptional terminators (stem-loop structures) followed by a promoter region (−10 and −35 elements) and a ribosome binding site (RBS) in 2 intergenic regions, between RGna_RS10350 and RGna_RS10345, and between RGna_RS10315 and RGna_RS10310. There was no co-occurence of distinct promoter and transcriptional terminator in other intergenic regions. This analysis suggests that the GH98 gene belongs to an operon of 7 genes comprising the genes encoding GH73 and GH95, while the GH31 gene appears to belong to a different operon (**Fig 8A**). In order to validate the in silico analysis, we analysed RNAseq data from *R*. *gnavus* ATCC 29149 grown on mucin or glucose (Glc) as sole carbon source [28]. RNAseq data showed induced transcription of genes RGna_RS10360 to RGna_RS10315 when *R*. *gnavus* ATCC 29149 was grown with mucin as compared to Glc, whereas a decreased in transcription was observed for RGna_RS10310 to RGna_RS10300 genes (**Fig 8B**). This analysis confirmed the in silico prediction that RGna_RS10310 to RGna_RS10300 are present on a different operon (**Fig 8A**).

To further determine the role of the GH98 operon in *R*. *gnavus* metabolism of blood group A antigens, the cDNA was prepared from RNA extracted from *R*. *gnavus* ATCC 29149 grown on BgA II, and a PCR was performed to amplify intergenic regions between genes RGna_RS10360 to RGna_RS10315. An amplicon was obtained for all the intergenic region between RGna_RS10360 and RGna_RS10315 (**S8 Fig**). This analysis suggests that the GH98 operon includes 10 genes, from RGna_RS10360 to RGna_RS10315 included, and not 7 as predicted in silico but supports the in silico prediction that both GH73- and GH95-encoding genes are part of the operon. It is worth noting that the GH98 gene operon is absent from the genome of the *R*. *gnavus* E1 strain, which is unable to grow on mucin [26], and no close homologues were found for the GH-encoding genes (RGna_RS10325, RGna_RS10330 and RGna_RS10315), suggesting the potential involvement of GH98, GH73, and GH95 in the ability of *R*. *gnavus* ATCC 29149 to degrade blood group A antigens found in mucin.

### *Rg*GH98 confers *R*. *gnavus* strains the ability to grow on mucin

In order to determine the contribution of *Rg*GH98 in the capacity of *R*. *gnavus* strains to grow on mucin, *R*. *gnavus* E1 and ATCC 29149 strains were grown under anaerobic conditions in mimimum medium supplemented with *Rg*GH98-treated or untreated mucin as sole carbon source. The effect of *Rg*GH98 treatment on mucin was first confirmed by MALDI-ToF MS following reductive β-elimination and permethylation. The MS analysis of untreated pPGM and *Rg*GH98-treated pPGM showed a high degree of fucosylation (approximately 38% and 38.6%, respectively) and very low level of sialylation (approximately 3.8% and 3.3%, respectively), in agreement with the mucin glycosylation profile of pPGM [37]. The glycan peak at 708 Da, corresponding to a trisaccharide composed of Fuc, Gal, and GalNAc increased from 6.6 nmol/mg of pPGM to 9.6 nmol/mg of pPGM following *Rg*GH98 treatment (**S9A Fig**). Fragmentation of this peak showed that the glycan structure bound on mucin in the untreated pPGM was Fuc-Gal-GalNAc, whereas, in the *Rg*GH98-treated pPGM, fragmentation of the 708 Da peak led to the appearance of a peak at 449 Da, corresponding to the fragment of reduced Fuc-Galactitol found in BgAtri (**S10B Fig**). In addition, a glycan peak corresponding to FucHexNAc4Gal3GalNAc that was found in the untreated pPGM dropped below detection levels following pPGM treatment with *Rg*GH98, although the presence of BgA in this structure could not be confirmed by MS/MS. The remaining fucosylated peaks showed a marginal reduction in abundance (**S9A Fig**). This analysis confirmed that the *Rg*GH98 treatment of pPGM led to the release of BgAtri, as previously shown by HPAEC-PAD (**Fig 4A**). The concentration of BgAtri released by *Rg*GH98 in the medium prior to the growth assay was estimated to be 6.08 ± 0.51 nmol/mg of pPGM by HPAEC-PAD (**S10 Fig**).

**Fig 9:**
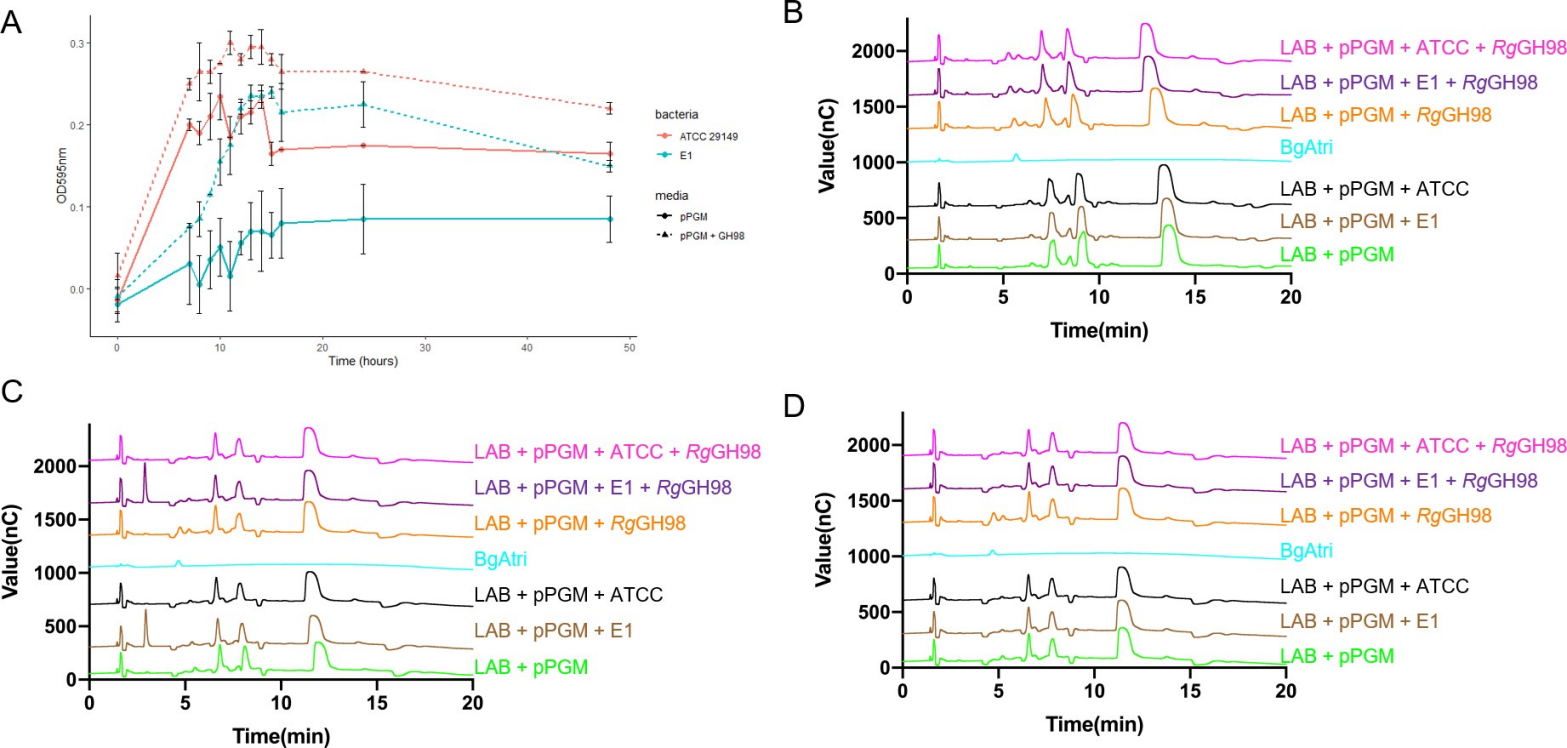
*R*. *gnavus* growth on mucin. (**A**) *R*. *gnavus* E1 and *R*. *gnavus* ATCC 29149 growth on untreated or *Rg*GH98-treated mucin, followed by HPAEC-PAD analysis of spent media at time = 0 h (**B**), time = 9 h **(C)**, time = 48 h **(D)**. Growth curves were performed using the LAB medium supplemented with *Rg*GH98-treated or untreated pPGM with *R*. *gnavus* E1 or ATCC 29149. Cultures were done in 2 biological replicates. BgAtri, LAB supplemented with pPGM and LAB supplemented with *R*gGH98-treated pPGM were used as controls in the HPAEC analysis. Underlying data can be found in S1 Data. BgAtri, BgA trisaccharide; HPAEC-PAD, high-pH anion exchange chromatography with pulsed amperometric detection; pPGM, purified pig gastric mucin.

Following *Rg*GH98 enzymatic treatment, *R*. *gnavus* E1 was able to grow on *Rg*GH98-treated pPGM at levels comparable to that of *R*. *gnavus* ATCC 29149 on untreated mucin. After 48 h, the *Rg*GH98 enzymatic treatment of mucin led to an overall increase in *R*. *gnavus* E1 and ATCC 29149 cell density as compared to untreated mucin, which was significant for *R*. *gnavus* E1 at 9 h (*p* = 0.023) and 48 h (*p* = 0.033) (**Fig 9A**), as also confirmed by qPCR analysis (**S11 Fig**). Growth of *R*. *gnavus* E1 and ATCC 29149 on *Rg*GH98-treated mucin led to the disappearance of the BgAtri peak after 9 h of growth (**Fig 9C**) as also shown after 48 h (**Fig 9D**), while the peak is detected at 0 h prior to growth (**Fig 9B**). In order to determine whether *R*. *gnavus* E1 could directly benefit from the released product of the *Rg*GH98-treated mucin, *R*. *gnavus* strains were grown on commercial BgAtri or BgA II as sole carbon source and the supernatant monitored by HPAEC-PAD during 28 h growth. Both *R*. *gnavus* ATCC 29149 and E1 strains could grow on commercial BgAtri as sole carbon source at 1.5 mM and 0.5 mM concentrations, whereas *R*. *gnavus* ATCC 29149 but not E1 could grow at 0.05 mM concentration (**S12A Fig**). Only ATCC 29149 could grow on BgA II (**S12B Fig**). The HPAEC-PAD analysis showed a decrease of the BgAtri peak (**S12C Fig**) or BgA II peak (**S12B Fig**) after 24 h and 10 h, respectively, while no Fuc peak could be detected. A comparative transcriptomics analysis of the complement of fucosidase genes encoded by *R*. *gnavus* ATCC 29149 and E1 genomes was carried out by quantitative reverse transcription PCR (RT-qPCR) to further investigate the metabolism of blood group A antigens by these bacteria when grown on BgA II and BgAtri, respectively. GH29-encoding gene RGna_RS05890 and GH95-encoding gene RGna_RS14395 were the highest fucosidase genes transcribed when *R*. *gnavus* ATCC 29149 was grown on BgA II (**S3A Table**), whereas GH29-encoding gene RUGNEv3_11127 and GH95-encoding gene RUGNEv3_40027 were mostly expressed when *R*. *gnavus* E1 was grown on BgAtri (**S3B Table**). Interestingly, RUGNEv3_40027 protein shows 94.5% aa identity with WP_004841212.1 (protein product of gene RGna_RS14395), which was shown in vitro to be active against BgAtri, the product of *Rg*GH98 enzymatic reaction **(S13 Fig**), suggesting that BgAtri may be further degraded by *R*. *gnavus* GH95 fucosidases RUGNEv3_40027 or WP_004841212.1 through cleavage of the Fucα1,2-linkage as part of its metabolism pathway although this will need further investigation.

Since we showed that *R*. *gnavus* E1 could only grow at BgAtri concentrations above those obtained from the *Rg*GH98-mucin treatment when used as sole carbon source, it is likely that the bacteria may also benefit from facilitated access to the underlying mucin glycan chain to sustain its growth. In order to test this hypothesis, growth media were collected after 24 h of culture on *Rg*GH98-treated and untreated mucin, and free and *O*-linked glycans were analysed by MS (**S9A Fig**). When *R*. *gnavus* E1 was grown on untreated pPGM, the abundance of di-fucosylated structures dropped below detection levels, and there was a reduction in glycans composed of FucGalGalNAc, FucHexNAc2Gal2GalNAc, and FucHexNAcGal2 (**S9A Fig**), which could contribute to the basal growth. However, when *R*. *gnavus* E1 was grown on pPGM pretreated with *Rg*GH98, there was a significant decrease in the abundance of glycans composed of FucHexNAc2Gal3GalNAc, FucHexNAc3Gal2GalNAc, and FucHexNAcGalGalANc (**S9A Fig**) as compared to growth on untreated pPGM, suggesting that this strain benefits from the underlying glycan structures after BgAtri release. No differences in the abundance of fucosylated glycans were detected between untreated and *Rg*GH98-treated pPGM media from the *R*. *gnavus* ATCC 29149 culture, as expected since *R*. *gnavus* ATCC 29149 expresses *Rg*GH98 (**S9A Fig**).

Together, these data support the role of *Rg*GH98 in supporting the growth of *R*. *gnavus* strains in mucin by releasing BgAtri that can be metabolised by *R*. *gnavus* E1 strain and by providing facilitated access to the underlying mucin glycan chain.

## Discussion

Gut bacteria have evolved to express a wide range of GHs with distinct ligand specificity, contributing to their fitness across nutritional niches [4,38]. *R*. *gnavus*, a human gut symbiont of the infant and adult microbiota [22–24], encodes GH33 sialidase [28,29,39] and GH29/95 fucosidases [30] active against mucin glycan epitopes [26]. Here, we showed that *R*. *gnavus* ATCC 29149 encodes a GH98 enzyme with specificity for BgA over BgB and BgH (O) antigens. The A and B antigens are derived from the H antigen through the action of an α-1,3-N-acetylgalactosamyl-transferase and α-1,3-galactosyltransferase, respectively, adding α-1,3 linked GalNac or Gal residues, respectively, at the nonreducing end of Fucα1-2Galβ1-4GlcNAc. The presence of these antigens in secretions, such as in intestinal mucins, is regulated by fucosyltransferase 2 (FUT2), which catalyses the transfer of Fuc to terminal Gal through α1,2 linkages substrates. About 80% of the population has a “nonsecretor” status and expresses blood group antigens in both the gastrointestinal (GI) tract and secretion. Many bacterial and viral pathogens exploit these host carbohydrate antigens for adherence as a precursor to colonisation or infection [40]. In addition, both the ABO types and the secretor status have been shown to affect the composition of the gut microbiota, although genome-wide association studies based on microbiome analysis of faecal samples have yielded conflicting results and information on the impact of blood group antigens on biogeographical communities in the gut is currently lacking [1,41–43]. Based on our preliminary analysis, the gene-encoding GH98 is present across 96% of genome-sequenced *R*. *gnavus* strains, while the GH98 operon occurs across 68% of the strains (**S6 Table**). The specificity of *R*. *gnavus* GH98 for BgA antigens may provide *R*. *gnavus* an advantage in colonising blood group A individuals with secretor status in the population. In line with a study showing that both secretor status and blood group antigen expression affect the Lachnospiraceae family of bacteria within the gut microbiome, with lower abundances noted in nonsecretors and higher abundances in secretors of various blood groups [42], it is tempting to speculate that the mucus-associated microbiota of blood group A individuals with secretor status may be enriched in GH98-expressing *R*. *gnavus* strains. In the gut, blood group A antigen substrates are most likely to be found in peripheral terminal epitopes of human intestinal mucins with variations along the GI tract. Although structural information is only available for a limited number of studies based on different methodologies, sample preparation and original material [6,7,9,44], it is possible that the presence of GH98 confers regio-selectivity to *R*. *gnavus* strains along the length of the colon, although this would need to be validated experimentally. The specificity of *R*. *gnavus* GH98 for BgA antigens may also influence *R*. *gnavus* strain acquisition in infants since human milk oligosaccharide (HMO) profile is determined by both secretor and Lewis (Le) status [45], and we previously showed that *R*. *gnavus* mucin-glycan foraging strains are able to consume HMOs [44]), perhaps contributing to the early adapatation of *R*. *gnavus* to the infant gut [24].

Although *Rg*GH98 shares the catalytic machinery of GH98 family members functionally characterised to date, its specificity to BgA appears unique to this enzyme, as also suggested by the SSN analysis. Both E-ABase from *C*. *perfringens* ATCC 10543 and *Sp*3GH98 from *S*. *pneumoniae* TIGR4 are capable of liberating the terminal trisaccharide, GalNAcα1-3(Fucα1–2)Gal) and Galα1-3(Fucα1–2)Gal from glycoconjugates containing BgA and BgB glycotopes, respectively. In contrast, *Sp*4GH98 from *S*. *pneumoniae* TIGR4 is active on the Lewis Y antigen, which is unique to this enzyme [14]. BgA and BgB antigens both contain the Galβ1-4GlcNAc glycosidic linkage targeted by GH98 enzymes but differ by the presence of GalNAc or Gal in the nonreducing end of BgA or BgB, respectively. We showed that *Rg*GH98 specificity for BgA is achieved through the precise positioning of amino acid side chains around the BgA GalNAc moiety, as shown in the crystal structure of the complex between *Rg*GH98 E411A and BgA II, principally through Gln 305, Trp 528, and Lys 788. Of note, Lys 788 is part of the C-term domain, supporting earlier alignment analyses suggesting a close spatial interaction of this domain with the catalytic domain across GH98 enzymes [31]. The importance of this residue was confirmed here by site-directed mutagenesis, revealing that the *Rg*GH98 K788A mutant lost enzymatic activity against BgA II while retaining its thermostability.

Transcriptomics analyses showed that *Rg*GH98 is part of an operon, which is up-regulated when *R*. *gnavus* ATCC 29149 is grown on mucin as sole carbon source. In line with the specificity of the purified *Rg*GH98 enzyme to BgA II, *Rg*GH98 was shown to be active on mucin, releasing BgAtri as confirmed by HPAEC and MS. Further, we showed that *R*. *gnavus* E1 was able to grow on *Rg*GH98-treated mucin and to metabolise the released BgAtri. No Fuc intermediate could be detected during growth of *R*. *gnavus* E1 or ATCC 29149 on BgAtri or BgA II, suggesting that *R*. *gnavus* may be able to transport and metabolise the trisaccharide or that Fuc is readily transported and consumed by the bacteria. According to the molecular cluster analysis, a predicted GH95 fucosidase (RGna_RS10315) as well as a GH73 (RGna_RS10330) with putative endo-β-N-acetylglucosaminidase specificity appear to be part of the GH98 operon and may also be involved in BgA II metabolism; also, this remains to be functionally demonstrated. In addition, our transcriptomics analyses revealed fucosidase candidates encoded by *R*. *gnavus* E1 or ATCC 29149 outside the GH98 operon, which may be involved in BgAtri metabolism. We demonstrated that the recombinantly expressed WP_004841212.1 GH95 fucosidase (which is highly similar to RUGNEv3_40027 from *R*. *gnavus* E1) could cleave off the α1–2 linkage in GalNAcα1–3[Fucα1–2]Galβ1-4GlcNAc, supporting their potential contribution to BgAtri metabolism. Further HPAEC and MS analyses showed that *R*. *gnavus* E1 also benefits from the uncapped mucin glycan chain, which becomes accessible to other mucin-glycan degrading GHs encoded by *R*. *gnavus* E1 genome including RUGNEv3_10180 (GH29), _10181 (GH95), _10623 (GH29), _10587 (GH95), _30029 (GH95), _30022 (GH20), and _30140 (GH20) [26]. Together, these data indicate that *Rg*GH98 confered *R*. *gnavus* E1 strain the ability to grow on mucins by enabling E1 to access the released BgAtri as well as accessing the underlying mucin glycan chain and further support the role of GHs in the adaptation of *R*. *gnavus* strains to distinct nutritional niches.

Blood group antigens can play a direct role in infection by serving as receptors and/or coreceptors for microorganisms, parasites, and viruses [40]. In addition to ABO, virus binding and host susceptibility are also heavily dependent on the secretor status [40]. For example, norovirus was found to bind to saliva from group O and A secretors but not to saliva from nonsecretors or group B individuals [46] (see for a review). Secretor and salivary ABO blood group antigen status may also contribute to the prediction of rotavirus vaccine protection [47]. Recently, clinical reports suggested the involvement of ABO blood groups in COVID-19 susceptibility with blood group A being associated with higher risk of of SARS-CoV-2 infection [48–53]. The specificity of *Rg*GH98 for blood group A antigen could therefore have potential application for diagnostics or therapeutics.

## Materials and methods

### Materials

All chemicals were obtained from Sigma (St Louis, MO, USA) unless otherwise stated. 2′-fucosyllactose (2′FL), 3-fucosyllactose (3FL) and 2′,3-difucosyllactose (DFL) and 6′-sialyllactose (6′SL) were from Glycom (Esbjerg N, Denmark). Lewis A (LeA), α1,3Gal-Lewis X (αGal-LeX), blood group A tetrasaccharide type I (BgA I), blood group A tetrasaccharide type II (BgA II), blood group A pentasaccharide type IV (BgA IV), blood group A tetrasaccharide type V (BgA V), GalNAcα1–3(Fucα1–2)Gal trisaccharide of the A antigen (BgAtri, used for ITC, STD NMR, and growth assays), blood group B tetrasaccharide type II (BgB II), blood group B pentasaccharide type IV (BgB IV), and blood group H trisaccharide (BgH) were from Elicityl (Crolles, France). Lewis X (LeX) and Lewis Y tetrasaccharide (LeY) were from Dextra Laboratories (Reading, UK). FA2G2 *N*-glycan was from Ludger (Oxford, UK). GlcNAc-*p*NP, Fucα1,6GlcNAc, and GalNAcα1–3(Fucα1–2)Gal trisaccharide of the A antigen (BgAtri, used in enzymatic assays) were from Carbosynth Limited (Campton, UK). Xyloglucan from tamarind seed and arabinoxylan from wheat were from Megazyme (Wicklow, Ireland). pPGM was obtained as previously described [54]. Blood group endo-β-galactosidase 98A from *S*. *pneumoniae* (*Sp*Bgg98A) was purchased from NZYTech (Lisbon, Portugal). Unconjugated *Ulex Europaeus* Agglutinin I (UEA I) was from 2BScientific Limited (Oxfordshire, UK). Recombinant fucosidase WP_004841212.1 (formerly RUMGNA_00842) from *R*. *gnavus* ATCC 29149 was produced in-house as previously reported [30].

### In silico analyses

The domain architecture of the putative GH98 in *R*. *gnavus* ATCC 29149 (WP_039959992.1) was analysed via InterProScan (InterPro 82.0). The analysed WP_039959992.1 protein sequence was 1,357 aa, but the presence of an alternative start codon could result in a protein 9 aa longer.

The cluster of genes surrounding the gene coding for the putative GH98 protein was analysed using the following in silico tools. Protein function prediction was based on automatic annotation and confirmed by BlastP [55] except for predicted GHs identified in our previous work [26]. Intergenic regions were analysed in silico: (i) Putative transcriptional terminators were predicted using the RNAfold programme (http://rna.tbi.univie.ac.at/cgi-bin/RNAfold.cgi) [56]; and (ii) prediction of bacterial sigma70 promoters was performed using the BPROM online tool [57]. When promoters were found, putative RBS were identified manually using the canonical Shine-Dalgarno sequence as a reference.

For SSN analysis, the sequences encoding GH98 enzymes were extracted from the the CAZy database (www.cazy.org) on 17 April 2020. The amino acid sequences were then used to generate a SSN using the Enzyme Function Initiative-Enzyme Similarity Tool (EFI-EST) [58]. After filtering sequences less than 250 aa from the CAZy database, a total of 355 GH98 sequences were analysed by SSN with an alignment score of 120. The SSN data were visualised using Cytoscape 3.6 [59].

Sequence alignments with functionally characterised GH98 enzymes were performed using Clustal Omega [60] and modular assignments established based on Interpro database (Interpro 80.0, 18 June 2020).

The occurrence of GH98 across genome-sequenced *R*. *gnavus* strains (84 to date) was carried out using Standard Nucleotide BLAST (BLASTN). The nucleotide sequence of GH98 gene, RGna_RS10325, was downloaded from NCBI’s Gene resources as a FASTA file along with the 10 genes that make up the GH98 operon, including RGna_RS10315, RGna_RS10320, RGna_RS10325, RGna_RS10330, RGna_RS10335, RGna_RS10340, RGna_RS10345, RGna_RS10350, RGna_RS10355, and RGna_RS10360. These 10 genes were concatenated into a single FASTA file. The nucleotide sequence of the GH98 gene and the concatenated FASTA file containing the nucleotide sequences of the GH98 operon were each submitted as query sequences in the BLASTN programme. Genomes of *R*. *gnavus* strains were downloaded as FASTA files from the NCBI Refseq Genomes FTP site and individually submitted as subject sequences in the BLASTN programme. The strain was considered positive for GH98 when the predicted operon or GH98 protein had a percent identity and percent query coverage of 80% and above.

### Cloning, expression, site-directed mutagenesis, and purification

*R*. *gnavus* ATCC 29149 genomic DNA was purified from the cell pellet of a bacterial overnight culture (1 mL) following centrifugation (5,000*g*, 5 min) using the GeneJET Genomic DNA Purification Kit (Thermo Fisher, UK) according to the manufacturer’s instructions. The sequence of *Rg*GH98 (44–946 aa) excluding the signal sequence, the C-term galactose-binding-like domain (GBLD) and C-term fibronectin type 3 (FN3) domain (see **Fig 1C**) was amplified by PCR and cloned into the pET-28a (+) vector (Novagen) using NdeI and XhoI restriction sites (New England Biolabs, USA). The *Rg*GH98 E411A mutant was produced by NZYTech (Lisbon, Portugal). The other *Rg*GH98 mutants, K788A, W528A, W528D, Q305A, and Q305W, were generated using the NZYMutagenesis kit (NZYTech, Portugal) according to the manufacturer’s instructions. The individual region encoding *Rg*GH98 N-term GBLD (Ala44-Gly272) and C-term domain (Val689-Val881) were amplified by PCR and cloned into pET-28a by in-fusion cloning (Takara, Japan). The catalytic and C-term domains (Cd-C-term, Phe253-Phe880) was amplified from *Rg*GH98 or E411A mutant by PCR and cloned into pET-28a using BamHI and XhoI restriction sites (New England Biolabs, USA). All constructs were designed to contain an N-terminal poly (x6) histidine tag (His_6_-tag). Primers used for PCR are shown in **S4 Table**. DNA manipulation was carried out in *E*. *coli* XL10-Gold cells (Stratagene, USA). Sequences were verified by DNA sequencing at Eurofins MWG (Ebersberg, Germany).

The recombinant proteins were expressed in *E*. *coli* Tuner (DE3) as previously described [30]. Briefly, *E*. *coli* Tuner (DE3) cells were cultured in LB broth to exponential phase (OD_600_ of 0.6) at 37°C and induced with 0.2 mM isopropyl-β-D-thiogalactopyranoside (IPTG). For the production of *Rg*GH98 and *Rg*GH98 mutants, E411A, K788A, W528A, W528D, Q305A, Q305W, and *Rg*GH98 N-term GBLD and C-term as well as RUMGNA_00842, cells were further cultured for 48 h at 16°C and later harvested by centrifugation at 7,000 × *g* for 10 min. The His-tagged proteins were purified by immobilised metal affinity chromatography (IMAC) and further purified by gel filtration (Superdex 75 and 200 columns) on an Akta system (GE Health Care Life Sciences, Little Chalfont, UK). For *Rg*GH98 Cd-C-term and *Rg*GH98 E411A Cd-C-term, cells were cultured overnight at 16°C and later harvested by centrifugation at 4,000 × *g* for 10 min and the recombinant proteins purified by IMAC using TALON resin (Takara Bio). The elution buffer contained 20 mM Tris–HCl, 150 mM NaCl (pH 8.0) with increasing amounts of imidazole from 10 mM to 100 mM. Protein purification was assessed by standard SDS–polyacrylamide gel electrophoresis using the NuPAGE Novex 4% to 12% Bis-Tris (Life Technologies, Paisley, UK). Protein concentration was measured with a NanoDrop (Thermo Scientific, Wilmington, USA) and using the extinction coefficient calculated by Protparam (ExPASy-Artimo, 2012) from the peptide sequence.

### Inductively coupled plasma mass spectrometry (ICP-MS) analysis

*Rg*GH98 (2.5 mg) was dialysed 72 h in ultrapure water. The dialysate was then freeze dried and 2.2 mg digested with ultrapure nitric acid and hydrogen peroxide. The digest was then diluted in Rhodium internal standard and Milli-Q water. The elemental content of the sample was determined using a Thermo TQ triple quad spectrometer with the following operating conditions: cooling flow rate: 14.0 L/min; auxillary gas flow rate: 0.8 L/min; sampling depth: 5 mm; additional gas flow: 75%; spray chamber: 2.7 degrees; nebuliser flow rate: 1.144 L/min; pump speed: 15 rpm; RF power: 15,550 W).

### Activity assays and kinetics

*Rg*GH98 (2 μM) was incubated with GlcNAc-*p*NP (16 mM) in 50 mM citrate buffer (pH 6.0) for 1 h. The reaction was stopped with 1.8 M Na_2_CO_3_ and the absorbance of *p*NP measured at 405 nm using a using a 96-well plate reader (BMG Labtech, Ortenberg, Germany).

To determine *Rg*GH98 substrate specificity, 1 μM enzyme was incubated with 100 μM oligosaccharides (2′FL, 3FL, DFL, LeA, LeX, αGal-LeX, LeY, Fuc1,6GlcNac, *N*-Acetyllactosamine (LacNAc), BgH, BgA I, BgA II, BgA IV, BgB II and BgB IV) or 1 mg/mL polysaccharide (xyloglucan and arabinoxylan) or 11.1 mg/mL pPGM in 50 mM citrate buffer at pH 6, 37°C for 24 h or 48 h for pPGM. A final concentration of 10 μM enzyme was used to test *Rg*GH98 activity against 5 ng/μL FA2G2. *Sp*Bgg98A from *S*. *pneumoniae* (NZYTech) was used as a control. The optimal pH were determined with 10 μM enzyme against 100 μM BgA II at 37°C for 30 min in 50 mM citrate buffer with pH 4.5 to 6.

To determine RUMGNA_00842 fucosidase activity, 1 μM enzyme was incubated with 100 μM BgAtri or 1 mg/mL of pPGM at 50 mM citrate buffer at pH 6, 37°C for 24 h.

All enzymatic assays were done at least in duplicates. Reactions were then heated at 95°C for 10 min before centrifuged at 17,000*g*, and supernatants were then analysed by high-pH anion exchange chromatography with pulsed amperometric detection (HPAEC-PAD) and/or liquid chromatography with fluorescence detection and mass spectrometric detection (LC-FD-MS/MS) as decribed below.

For kinetics, *Rg*GH98 (10 μM) was incubated in 50 mM citrate buffer (pH 5) at 37°C for 30 min with BgA II at concentration varying from 25 μM to 4 mM and the activity measured at 5 min interval for each time point. The reactions were stopped by heating at 95°C for 5 min and analysed by HPAEC-PAD. Fuc (20 μM) were added as internal standard to facilitate quantification. A standard curve was made with a range of GlcNAc from 5 to 100 μM containing 20 μM of Fuc. Kinetic parameters were calculated based on the Michaelis–Menten equation using a nonlinear regression analysis programme (Prism 5, GraphPad, San Diego, USA).

For HPAEC-PAD analysis, the samples were centrifuged at 17,000 × *g* and the supernatant analysed on a Dionex ICS5000 system (Thermo Scientific, Hemel Hempstead, UK). The sugars were separated on a CarboPac PA1 column protected with a guard column using the following gradient conditions: 0 min, 18 mM NaOH; 20 to 35 min, 100 mM NaOH; 35.1 to 50 min, 18 mM NaOH.

For quantification of BgAtri released from pPGM, a series of BgAtri standards 6.25 μM, 12.5 μM, 25 μM, 50 μM, 75 μM, and 100 μM were used to generate the standard curve and calculate the slope (nC*min/μM), the amount of BgAtri was determined using the linear calibration. Experiments were performed in duplicates.

For LC-FD-MS/MS analysis, the reactions were stopped by heating 95°C for 5 min and then dryed down using Savant SpeedVac centrifugal evaporator (Thermo Fisher, Wilmington, USA), labelled at the reducing end with procainamide using the glycan labelling kit with sodium cyanoborohydride reductant (Ludger, Oxford, UK), and purified using S-cartridges (Ludger, Oxford, UK) to remove the excess dye. The samples were dried by speed vacuum and resuspended in 50 μL of acetonitrile:water solvent. The suspensions were then injected onto a Waters ACQUITY UPLC Glycan BEH amide column (2.1 × 150 mm, 1.7 μm particle size, 130 Å pore size) at 40°C on a Dionex Ultimate 3000 UHPLC instrument with a fluorescence detector (λ_ex_ = 310 nm, λ_em_ = 370 nm) coupled to a Bruker Amazon Speed ETD. A 50-mM ammonium formate solution (pH 4.4) (Ludger, Oxford, UK) was used as mobile phase A and acetonitrile (Romil, UK) was used as mobile phase B. A 70-min gradient was used with mobile phase B from 70% to 62% for FA2G2, 85% to 65% for BgA II/BgB II from 0 to 53.5 min at a flow rate of 0.4 mL/min followed by mobile phase B from 51% to 0% from 53.5 min to 55.5 min at flow rate of 0. 2 mL/min, and 2 min stabilisation, mobile phase B from 0% to 70% from 57.5 min to 59.5 min at a flow rate 0.2 mL/min, and then last for 6 min, from 65.5 min to 66.5 min, the flow rate was changed back to 0.4 mL/min and then equilibrated for 3.5 min.

### Differential scanning fluorimetry (DSF) assay

DSF was used to analyse the thermostability of *Rg*GH98 and *Rg*GH98 K788A, W528A, W528D, Q305W, and Q305A. A volume of 4 μL of protein sample (1 mg/mL) in 20 mM Tris–HCl, 150 mM NaCl (pH 7.9) was mixed with 2 μL 500 mM citrate buffer (pH 5), 2 μL SYPRO orange (100×), and made up to 20 μL with milliQ water. The measurement was performed in StepOnePlus PCR instrument following the programme: start temperature 25°C; end temperature 80°C; temperature slope 2.0°C/min. The Tm was determined from the minimum of the plot of derivative fluorescent-based signal against temperature.

### Glycan arrays

Recombinant His6-tagged *Rg*GH98 E411A, *Rg*GH98 C-term, and *Rg*GH98 N-term GBLD were expressed and purified as described above. Three protein concentrations (5, 50, and 200 μg/mL) were prepared in binding buffer (20 mM Tris–HCL (pH 7.4), 150 mM sodium chloride, 2 mM calcium chloride, 2 mM magnesium chloride, 0.05% Tween 20, 1% BSA) and screened for binding to Core H glycan microarray glycans at the CFG. Version 5.4 of the printed array consists of 585 glycans in replicates of 6. Protein samples were detected on the glycan array by fluorescence-labelled primary antibody against His tag. The scanner response is linear to a maximum RFU value of about 50,000. The data were sorted by RFU (high to low) and the highest and lowest point from each set of 6 replicates removed to eliminates some of the false hits that contain a single very high or low point. The average RFU value (of 4 replicates), the standard deviation, and %CV (%CV = 100 X Std. Dev / Mean) for each protein tested was provided by the CFG and the graph of glycan number versus average RFU with standard error of the mean (SEM) plotted in the error bars (standard deviation/2 = SEM).

### Isothermal titration calorimetry (ITC)

ITC experiments were performed using the PEAQ-ITC system (Malvern, Malvern, UK) with a cell volume of 200 μL. Prior to titration, *Rg*GH98 E411A were exhaustively dialysed into 50 mM citrate buffer (pH 5). The ligand was dissolved in the dialysis buffer. UEA I and the ligand thereof were dissolved in 10 mM HEPES buffered saline (pH 7.5), 0.1 mM CaCl_2_.

For *Rg*GH98 E411A, the cell protein concentration was 260 μM, and the syringe ligand was 5 mM for all ligands tested. For UEA I, the cell protein concentration was 100 μM, and the syringe ligand concentration was 5 mM for all ligands tested. Three controls with titrant (sugar) injected into the buffer, buffer injected to protein, buffer injected into buffer, were subtracted from the data. The analysis was performed using the Malvern software, using a single-binding site model. Experiments were carried out in triplicate for *Rg*GH98 E411A and duplicate for UEA I.

### Crystallisation and structure resolution

Crystallisation experiments were characterised by diffraction using the VMXi in situ beamline at Diamond Light Source prior to cryocooling crystals from the best conditions [61]. Apo crystals were grown in 20% PEG 500 MME, 10% PEG20K, 0.1 M sodium HEPES/MOPS (pH 7.5) in sitting drop vapour diffusion plates using *Rg*GH98 wild-type protein. Crystals for the BgAtri complex were grown in 12.5% MPD; 25% PEG 1000; 12.5% PEG 3350, 0.1 M sodium HEPES/MOPS (pH 7.5), 30 mM magnesium chloride hexahydrate, and 30 mM calcium chloride dihydrate using *Rg*GH98 wild-type protein. Crystals for the BgA II complex were grown in 20% PEG 500 MME, 10% PEG20K, 0.1 M (pH 6.5) imidazole/MES monohydrate (acid), 30 mM sodium fluoride, 30 mM sodium bromide, and 30 mM sodium iodide using the *Rg*GH98 E411A mutant protein. All drops were set up at 10 mg/mL with a starting protein volume of 0.15 μL and reservoir volume of 0.3 μL. Soaks were short, approximately 1 min with 10 mM compound. A 10% ethylene glycol solution was introduced to aid with cryoprotection. Crystals in the presence of compound were cryocooled once changes in crystal morphology were observed. Diffraction experiments were performed using beamlines i03 and i24 at Diaamond Light Source at a wavelength of 0.9763 Å and 0.97628 Å, respectively. Data were processed using Dials, Xia2, and Aimless [62–64]. Molecular replacement was performed using MOLREP and pdb 4D6C within the CCP4 package [34,65,66]. Two molecules were found in the asymetric unit. Automated model building was performed using BUCCANEER [67] followed by iterative cycles of refinement using using coot, REFMAC, and PDBredo [68–70]. Model validation was performed using Molprobity [71].

### Saturation transfer difference nuclear magnetic resonance (STD NMR) analysis

For STD NMR, all proteins were buffer exchanged using an amicon centrifuge filter unit with a 10-kDa MW cutoff in 25 mM d19-2,2-bis(hydroxymethyl)-2,2′,2″-nitrilotriethanol pH* 7.4 (uncorrected for the deuterium isotope effect on the pH glass electrode) D_2_O buffer and 50 mM NaCl. The ligands were dissolved in 25 mM d19-2,2-bis(hydroxymethyl)-2,2′,2″-nitrilotriethanol pH* 7.4, 50 mM NaCl. Ligand NMR assignment was performed by acquiring a set of 2D experiments (COSY, HSQC, TOCSY) using 1 mM ligand. To detect binding and for binding epitope determination, a concentration of 25 μM was used for each protein domain (*Rg*GH98 E411A, *Rg*GH98 N-term GBLD, *Rg*GH98 C-term) and 1 mM for the ligands. The on- and off-resonance spectra were acquired using a train of 50 ms Gaussian selective saturation pulses using a variable saturation time, with on-resonance frequency at 0.0 ppm and off-resonance frequency at 40 ppm. Binding experiments were performed at 2-s saturation time. Residual protein resonances were filtered out using a T_1rho_ filter of 40 ms. The STD NMR experiments were performed with a spectral width of 10 kHz and 32,768 data points using 256 or 512 scans on a Bruker Avance 800.23 MHz at 278 K.

Binding epitope mapping was obtained from the initial slopes of STD build-up curves (incremental saturation time from 0.5 to 5 s), calculated by performing a least-squares fitting of the following mono-exponential curve:

$$STD(t_{sat})=STD_{max}\_(1‐exp(-k_{sat}\cdot t_{sat}))$$

where STD(t_sat_) is the STD intensity for a saturation time t_sat_, STD_max_ is the maximum STD intensity, and k_sat_ is the rate constant for saturation transfer. In the limit t_sat_ →0 (initial slope, STD_0_):

$$STD_{0}=STD_{max}\cdot k_{sat}.$$

Importantly, STD_0_ gives a value that is independent of any relaxation or rebinding effects, allowing for an accurate binding epitope mapping determination. To this end, every value of STD_0_ was normalised against the proton with the largest intensity to give values in the range of 0% to 100%, which were then mapped onto the ligand structure.

For competition STD NMR experiments between BgA II and BgB II, STD NMR experiments (t_sat_ 2 s, 512 transients) were first run on 50 μM *Rg*GH98 E411A in the presence of 1 mM BgB II, and in a second experiment, 1 mM BgA II was added to the reaction, monitoring the displacement of BgB II by the intensity reduction on isolated well-resolved STD NMR signals of BgB II.

### *Rg*GH98 mucin treatment in vitro

Purified pPGM (10 mg/mL) was incubated with *Rg*GH98 (2 μM) in 50 mM citrate buffer (pH 5.0) for 24 h or 48 h. The reaction was stopped by heating at 50°C for 5 min, a reaction volume (0.2 mL) containing 2 mg of digested pPGM analysed by HPAEC-PAD, the rest was dialysed against water using a 7-kDa membrane. The samples inside the dialysis cassette (containing the treated pPGM) were recovered and freeze dried and the dialysate (containing the enzymatically released oligosaccharides) were concentrated by complete distillation and recovered with deionised water, following by desalting with a graphitised carbon column (SupelcleanTM ENVITM-Carb SPE Tubes (Pennsylvania, USA)), eluted in 10% acetonitrile with 0.1% trifluoroacetic acid and in 25% acetonitrile with 0.1% trifluoroacetic acid, freeze dried then dissolved in dionised water before analysing by HPAEC-PAD and MALDI-ToF MS.

### *R*. *gnavus* growth assays

*R*. *gnavus* ATCC 29149 [72] and E1 [73] strains were grown in an anaerobic cabinet (Don Whitley, Shipley, UK) in 14 ml tubes or in a 96-well plate, at 37°C with 85% N_2_, 10% H_2_, and 5% CO_2_. The growth was monitored by spectrophotometry with an Ultrospec 10 cell density metre from GE Healthcare (Chicago, IL, USA) at 600 nm or an Infinite F50 plate reader from Tecan (Männedorf, Zürich, Switzerland) at 595 nm.

Starter cultures were grown in BHI-YH as previously described [26]. Growth on single carbon sources utilised a minimum medium, either the semidefined YCFA medium [74] or the defined LAB medium [75] as indicated.

For growth assays with pPGM, 10 g/L pPGM was added to the medium and autoclaved. When pPGM was pretreated with *Rg*GH98, the recombinant enzyme was diluted in LAB medium, filter sterilised, and added to the LAB+pPGM medium at a final concentration of 1.3 μM 48 h before inoculation with the bacteria.

For growth assays with Glc, Fuc, or BgAtri, filter-sterilised stock solutions of the sugars were added to the LAB medium at a final concentration of 0.5% (w/v), 0.46% (w/v), and 0.09% (w/v), respectively.

### MALDI-ToF MS analysis of mucin glycosylation from growth cultures

Spent growth media (100 μL containing 1 mg of pPGM) were used for glycan analysis. 6′SL was used as internal standard at 10 μg/mg of pPGM. Bound glycans were released from mucins by reductive β-elimination in NaBH_4_ 0.5 M dissolved in NaOH 50 mM at 45°C for 16 h. Free glycans in the growth media were also reduced during this process. The reaction was quenched with dropwise addition of glacial acetic acid. The samples were desalted on an in-house prepared column of DOWEX 50W x8 H^+^ cation exchange resin, and borate was removed by coevaporation with methanol under nitrogen. The dried glycans were permethylated by the addition of 200 μL DMSO, 300 μL of NaOH base in DMSO (prepared as described in [76] and 150 μL iodomethane under vigorous shaking for 30 min at room temperature. The reaction was quenched by addition of 1 mL of H_2_O and excess of iodomethane was removed under nitrogen. Permethylated glycans were purified on SWIFT HLB cartridges (Sigma, St Louis, MO, USA), where contaminating salts were removed with H_2_O and permethylated glycans eluted with methanol. The permethylated samples were analysed by MALDI-ToF MS on a Bruker Autoflex (Bruker Daltonics). Peaks corresponding to glycans with a signal-to-noise ratio >3 were considered for the analysis. Two technical replicates from 2 biological replicates were carried out in total.

### Bacteria quantification by qPCR

Cells from a 2-mL aliquot of culture were harvested by centrifugation (10,000*g*, 5 min, 4°C), at different times of growth (0 h, 9 h, and 48 h). The cell pellet was kept frozen at −20°C until DNA extraction. The DNA extraction was carried out using Gene JET Genomic DNA Purification kit (Thermo Fisher Scientific) following supplier’s procedure for gram-positive bacteria, except for the elution step, which was performed with 50 μL of EB buffer instead of 200 μL. DNA quality and quantity were assessed using the NanoDrop 2000 spectrophotometer (Thermo Fisher Scientific) and Qubit dsDNA HS assay on Qubit 2.0 fluorometer (Thermo Fisher Scientific). Dilutions at 1 ng/μL were prepared in water containing 5 μg/ml Salmon Sperm DNA (Sigma-Aldrich).

The standard was a PCR fragment obtained by amplification of *R*. *gnavus* ATCC 29149 16S rRNA gene, as previously described in [77]. Briefly, the PCR was carried out using the HotStarTaq *Plus* Master Mix Kit (Qiagen) according to the supplier’s advice. A total of 35 cycles of 3 steps were performed with an optimised annealing temperature for the primers used (**S4 Table**) and a 2-min extension, following the supplier’s instructions. The PCR product was purified, quantified, and diluted in water to a concentration of 16.4 ng/uL, which equals to 10^10^ copies/μL. A series of 10-fold or 20-fold dilutions was then performed from 10^10^ copies/μL to 10^2^ copies/2 μL using 5 μg/mL salmon sperm DNA.

The qPCR was carried out in an Applied Biosystems 7500 Real-Time PCR system (Life Technologies) with qPCR primers targeting specifically *R*. *gnavus* 16S rRNA gene (**S4 Table**). Each qPCR reaction (10 μL) was performed in triplicates with 2 μL of DNA matrix (standards at 10^2^ copies/2 μL to 10^7^ copies/2 μL or DNA at 1 ng/μL) and 0.2 μM of each primer using the QuantiFast SYBR Green PCR kit (Qiagen) according to supplier’s advice (except for the combined annealing/extension step, which was extended to 35 s instead of 30 s). The standard curves showed a linear relationship of log input gene copy number versus the threshold cycle (C_T_), with acceptable values for the slopes and the regression coefficients (R^2^). The dissociation curves were also performed to check the specificity of the amplicons.

Gene copy number/mL of culture was calculated in each sample using the Ct value obtained, the standard curve equation (Ct = a × Log(gene copies/well) + b) and the amount of DNA extracted/mL of culture in the sample (β): gene copies/mL of culture = (β × 10((Ct − b)/a)) / 2.

### RNA extraction from *R*. *gnavus* ATCC 29149 growth cultures

Total RNA was extracted from 3 to 5 mL of mid- to late exponential phase cultures of *R*. *gnavus* in YCFA supplemented with either 0.5% Glc or 10 g/L pPGM or LAB supplemented with 1 mM of BgAtri or BgA II. Four biological replicates were performed for each carbon source. The RNA was stabilised prior to extraction by adding 1/5 vol of phenol (pH 4.3): ethanol (1:9) mixture to 1 vol of culture then incubating 30 min on ice and centrifuging for 5 min at 10,000*g* at 4°C. Cell pellets were stored at −80°C before extraction. Extraction was performed using phenol and chloroform as previously described [78]. Genomic DNA contamination was removed by DNAse treatment using TURBO DNA-free kit (Life Technologies, Paisley, UK) according to supplier’s recommendations.

The purity, quantity, and integrity of the DNase-treated RNA were assessed with NanoDrop 2000 Spectrophotometer, with Qubit HS RNA assay on Qubit 2.0 Fluorometer and with Agilent RNA 600 Nano kit on Agilent 2100 Bioanalyzer or with High-Sensitivity RNA ScreenTape on Agilent 4200 TapeStation (Agilent Technologies, Stockport, UK).

### RNAseq transcriptomics analysis

Sequencing of RNA extracted from *R*. *gnavus* ATCC 29149 grown in Glc or pPGM was previously described [28]. To compare the transcript expression levels across samples, the RNAseq reads were mapped onto the *R*. *gnavus* ATCC 29149 genome with the open source tool Bowtie v0.12.9 [79] using default parameters. Raw counts were normalised to the effective library size. Log2 (fold change) of gene transcription was calculated for each gene when ATCC 29149 was grown on mucin as compared to Glc.

### RT-qPCR transcriptomics analysis

DNAse-treated RNA (100 ng to 1 μg) from *R*. *gnavus* ATCC 29149 grown on BgA II and *R*. *gnavus* E1 grown on BgAtri was converted into cDNA using QuantiTect Reverse Transcription (RT) kit (Qiagen) according to supplier’s advice (including the genomic DNA elimination step). An RT negative control (RT−) was generated using the same amount of DNAse-treated RNA and following the same procedure but without addition of the reverse transcriptase.

qPCR was carried out in an Applied Biosystems 7500 Real-Time PCR system (Life Technologies). One pair of primers was designed for each target gene using ProbeFinder version 2.45 (Roche Applied Science, Penzberg, Germany) to obtain an amplicon of around 60 to 200 bp long. The primers were between 18 and 23 nt-long, with a Tm of 59 to 60°C (**S4 Table**). Calibration curves were prepared in triplicates for each pair of primers using 3- or 4-fold serial dilutions of *R*. *gnavus* genomic DNA. The standard curves showed a linear relationship of log input DNA versus the threshold cycle (CT), with acceptable values for the slopes and the regression coefficients (R^2^). The dissociation curves were also performed to check the specificity of the amplicons. Each qPCR reaction (10 μL) was then carried out in triplicates with 1 to 2 μL of a 5- or 10-fold diluted sample (cDNA or RT−) and 0.2 mM of each primer, using the QuantiFast SYBR Green PCR kit (Qiagen) according to supplier’s advice (except that the combined annealing/extension step was extended to 35 s instead of 30 s).

Data obtained with cDNA were analysed when CT values above 35 were obtained for the corresponding RT−. For each cDNA sample, the level of transcription of each gene tested was expressed as equivalent of gDNA concentration using the standard curve equation and the genes were ranked accordingly.

### PCR analysis of cluster

A PCR was performed with the cDNA (and RT negative control) from *R*. *gnavus* ATCC 29149 grown on BgA II to amplify fragments within the *Rg*GH98-encoding gene (RGna_RS10325) as well as fragments corresponding to the intergenic regions between genes RGna_RS10360 and RGna_RS10315. The primer sequences and the expected amplicon sizes are presented in **S4 Table**. Each PCR reaction (10 μL) was carried out with 0.4 μL to 1 μL of cDNA and 0.25 mM of each primer, using the HotStarTaq *Plus* Master Mix Kit (Qiagen) according to the supplier’s advice. Control reactions were also prepared by substituting the cDNA with water (negative control), 50 ng of *R*. *gnavus* ATCC 29149 genomic DNA (positive control) or RT negative control (gDNA contamination control). A total of 35 cycles of 3 steps were performed with an annealing temperature of 60°C and a 3-min extension following the supplier’s instruction. PCR fragments were analysed by electrophoresis on a 1% agarose gel using the Midori Green Direct DNA Stain (Geneflow, UK).

### Statistical analyses

For the statistical analysis of *R*. *gnavus* growth assays on *Rg*GH98-treated versus untreated pPMG, the effect of *Rg*GH98 on growth was tested using 3 linear mixed models, with delta-OD at 9, 24, and 48 h (delta-OD is the OD value minus the OD of the corresponding “no bacteria” control) used as the outcomes, respectively, and the main effects of pPMG, *R*. *gnavus* strain, and their interaction as predictors. The delta-OD at 0 h was included as a covariate to account for baseline differences along with a random effect of biological replicate. Models were estimated using the lmerTest version 3.1–3 with lme4 version 1.1–27.1 packages for R version 4.1.1. Using these models, the effect of RgGH98 on OD at each period, stratified by strain, was estimated using the emmeans package version 1.6.3 for R.

For the analysis of qPCR data, growth was analysed using a linear mixed model. Three technical replicates (measurements) were available at each time point. Estimated concentrations were transformed onto a logarithmic scale, then the concentration was modelled using the interaction of time, strain, and enzyme and all of their 2- and 3-way interactions as predictors, with nested random effects corresponding to biological replicate and time within biological replicate. Models were estimated using lmerTest/lme4 for R as above, and the effects of enzyme on growth between 0 and 9 and between 0 and 48 h for each strain was calculated from each model using emmeans [80–83].

## Supporting information

S1 Table ICP-MS analysis of *Rg*GH98 metal ion content. ICP-MS, inductively coupled plasma mass spectrometry.
(XLSX)

S2 Table ITC thermodynamics parameters of UEA1 and *Rg*GH98 against BgH and BgA II, respectively. BgA II, blood group A tetrasaccharide type II; BgH, blood group H trisaccharide; ITC, isothermal titration calorimetry.
(XLSX)

S3 Table Transcriptomics analysis of fucosidase genes (and *Rg*GH98 gene for ATCC 20149) in *R*. *gnavus* strains grown on blood group A antigens. (**A**) *R*. *gnavus* ATCC 29149 was grown on BgA II. (**B**) *R*. *gnavus* E1 was grown on BgAtri. BgA II, blood group A tetrasaccharide type II; BgAtri, BgA trisaccharide.
(XLSX)

S4 Table Primers used in the study. (XLSX)

S5 Table Chemical shifts assignments and STD initial slopes of BgA II. BgA II, blood group A tetrasaccharide type II; STD, saturation transfer difference.
(XLSX)

S6 Table Presence or absence of GH98-encoding gene and operon across genome-sequenced *Ruminococcus gnavus* strains. (XLSX)

S1 Fig HPAEC-PAD analysis of *Rg*GH98 against fucosylated oligosaccharides and polysaccharides. 2′FL (**A**), 3FL (**B**), DFL (**C**), Fucα1,6GlcNAc (**D**, the upper panel is with *Rg*GH98 and the lower panel is with *Sp*GH98), xyloglucan (**E**), arabinoxylan (**F**). DFL, difucosyllactose; Fuc, fucose; HPAEC-PAD, high-pH anion exchange chromatography with pulsed amperometric detection; 2′FL, 2′-fucosyllactose; 3FL, 3-fucosyllactose.
(TIF)

S2 Fig Enzymatic characterisation of *Rg*GH98. (A) Optimal pH analysis of *Rg*GH98. The initial velocity against BgA II (100 μM) was measured at different pH values in 50 mM citrate buffer with the recombinant enzyme (10 μM) at 37°C. (B) Michaelis–Menten curve of *Rg*GH98 towards BgA II. The experiment was carried out in duplicates for optimal pH analysis and triplicates for Michaelis–Menten curve. BgA II, blood group A tetrasaccharide type II.
(TIF)

S3 Fig Sequence alignment of *Rg*GH98 with characterised GH98s from the CAZy database. Black triangle, Q305; circle, W528; diamond, K788.
(TIF)

S4 Fig Structural comparison with homologous GH98s. (**A**) *Rg*GH98 apo structure (wheat) aligned to *Rg*GH98 bound to BgAtri (Cd in green, and C-term domain in light cyan) bound structure. Fuc is coloured in pink, galactose in orange, and GalNAc in yellow. Amino acid identifiers refer to *Rg*GH98. (**B**) *Rg*GH98 bound to BgAtri aligned to *Sp*3GH98 (grey) bound to the same carbohydrate. Amino acid identifiers refer to *Sp*3GH98. (**C**) *Rg*GH98 bound to BgAtri aligned to *Sp*4GH98 (pink). Amino acid identifiers to *Sp*4GH98. The absence of the Trp 512 loop present in *Sp*4GH98 allows binding of BgA II by *Rg*GH98. (**D)** *Rg*GH98 bound to BgAtri (orange) aligned to *Rg*GH98 E411A mutant (green) bound to BgA II, highlighting steric clash between BgAtri bound Glu411 rotamer and BgA II. For clarity, only the tetrasaccharide carbohydrate is shown. (**E**) *Rg*GH98 E411A mutant (green) bound to BgA II aligned to *Sp*3GH98 (grey) bound to BgA II to highlight differing GlcNAc positioning. First residue number refers to *Rg*GH98 and second to *Sp*3GH98. **(F)** Magnesium and **(G)** calcium binding sites as observed in the *Rg*GH98 E411A with BgA II bound crystal structure. Fo-Fc (grey mesh) and anomolous difference omit maps are shown for both ions, with grey mesh for the Fo-Fc and orange mesh for the anomalous signal. Confirming that only one site is occupied by calcium. BgA II, blood group A tetrasaccharide type II; BgAtri, BgA trisaccharide; Cd, central/catalytic domain; C-term, C-terminal; Fuc, fucose; GalNAc, N-acetylgalactosamine; GlcNAc, N-acetylglucosamine.
(TIF)

S5 Fig Analysis of recombinant *Rg*GH98 variants. (**A**). SDS-PAGE analysis of purified *Rg*GH98 wt and specificity mutants. (**B**) HPAEC-PAD of *Rg*GH98 wt and mutants. Around 10 μM of recombinant enzyme was incubated with BgA II (0.1 mM) in 50 mM citrate buffer (pH 5) at 37°C for 24 h. Reactions were then heated at 95°C for 10 min before centrifugation at 16,000*g*, and supernatants were analysed by HPAEC-PAD. Experiments were done in triplicates. (**C**) DSF analysis of *Rg*GH98 wt and mutants. BgA II, blood group A tetrasaccharide type II; DSF, differential scanning fluorimetry; GlcNAc, N-acetylglucosamine; HPAEC-PAD, high-pH anion exchange chromatography with pulsed amperometric detection; wt, wild-type.
(TIF)

S6 Fig STD NMR analysis of *Rg*GH98 domains to BgA II. STD NMR binding experiments were performed at 2-s saturation time, with selective protein irradiation at 0.0 ppm. (**Left panel**) (**A**) Reference spectrum: BgA II/*Rg*GH98 E411A sample. (**B**) STD NMR spectrum of BgA II/*Rg*GH98 E411A. (**C**) STD NMR spectrum of BgA II/*Rg*GH98 N-term GBLD (no binding detected). (**D**) STD NMR spectrum of BgA II/ *Rg*GH98 C-term (no binding detected). Ligand chemical shifts assignments in S5 Table. (**Right panel**) (**A**) Reference spectrum: BgH/*Rg*GH98 E411A sample. (**B**) STD NMR spectrum of BgH/*Rg*GH98 E411A (no binding detected). (**C**) Reference spectrum: BgH and BgA II/*Rg*GH98 E411A sample (addition of BgA II to the sample in C). (**D**) STD NMR spectrum of BgH and BgA II/*Rg*GH98 E411A sample (binding from BgA detected). BgA II, blood group A tetrasaccharide type II; C-term, C-terminal; N-term GBLD, N-terminal galactose-binding-like domain; STD NMR, saturation transfer difference nuclear magnetic resonance spectroscopy.
(TIF)

S7 Fig Glycan array analysis of *Rg*GH98 variants. The recombinant proteins (**A)** *Rg*GH98 E411A, (**B)** N-term GBLD, and (**C**) C-term were screened on the CFG glycan array in 6 replicates. The highest and lowest point from each set of 6 replicates has been removed so the average is of 4 values rather than 6. The panels on the left show the overall binding events of *Rg*GH98 variants against 585 ligands. The panels on the right list the glycan compositions of the top 3 hits. Underlying data can be found in S1 Data. CFG, Consortium for Functional Glycomics; C-term, C-terminal; N-term GBLD, N-terminal galactose-binding-like domain; RFU, relative fluorescence unit.
(TIF)

S8 Fig Confirmation of the operonic structure of the GH98 gene cluster in *R*. *gnavus* ATCC 29149. A 2-step RT-PCR was performed on RNA extracted from *R*. *gnavus* ATCC 29149 grown on BgA II using primers targeting every intergenic region between genes RGna_RS10360 and RGna_RS10315, and the PCR products analysed by electrophoresis on agarose gel. Intergenic region between RGna_RS103XX and RGna_RS103YY is labelled XX/YY on the gel. PCR from RT negative control (RT−) was performed to confirm the absence of genomic DNA contamination of the RNA sample prior to RT. PCR negative (−) and positive (+) controls were carried out with water or ATCC 29149 genomic DNA as template, respectively. The sequences of the primers are provided in S4 Table. M, DNA ladder size marker (with increments indicated in base pairs). RT-PCR, reverse transcription PCR.
(TIF)

S9 Fig MS analysis of mucin in growth culture. (**A**) Quantification of fucosylated glycans from growth media supplemented with either untreated (green) or *Rg*GH98-treated (red) pPGM, in the presence or absence of *R*. *gnavus* strains. The bold composition “FucGalGalNAc” corresponds to the glycan peak at 708 Da and is indicative of either reduced BgAtri or Fuc-Gal-GalNAc-ol. (**B**) Fragmentation spectra of the pPGM glycan peak at 708 Da, centred around 470 Da. Fragments characteristic of BgAtri could only be found in the samples with *Rg*GH98-treated pPGM. For glycan analyses, datasets from 2 biological replicates with 2 technical replicates were used. Underlying data can be found in S1 Data. BgAtri, BgA trisaccharide; MS, mass spectrometry; pPGM, purified pig gastric mucin.
(TIF)

S10 Fig Quantification of BgAtri released by *R*gGH98 in the medium via HPAEC-PAD. (**A**) Released BgAtri from the medium prior growth assays along with standards. The experiment was carried out in 3 biological replicates (**B**) Standard curve was performed in triplicates. The amount of BgAtri released was calculated to be 66.9 μmol/L as average of 4 replicates (67.4 μmol/L, 65.7 μmol/L, 74.1 μmol/L, and 60.5 μmol/L). Underlying data can be found in S1 Data. BgAtri, BgA trisaccharide; HPAEC-PAD, high-pH anion exchange chromatography with pulsed amperometric detection.
(TIF)

S11 Fig Quantification of *R*. *gnavus* growth in mucin by qPCR. The results show the number of *R*. *gnavus* 16S rRNA gene copies/mL in the no-bacteria controls (left panel), in *R*. *gnavus* ATCC 29149 cultures (middle panel), and in *R*. *gnavus* E1 cultures (right panel) following 48-h growth on *Rg*GH98-treated mucin or untreated mucin. The qPCR analysis was carried out from 2 biological growth cultures in triplicates. The error bars correspond to the standard errors. Underlying data can be found in S1 Data. pPGM, purified pig gastric mucin; qPCR quantitative PCR.
(TIF)

S12 Fig Growth of *R*. *gnavus* strains on BgA antigens. *R*. *gnavus* E1 and *R*. *gnavus* ATCC 29149 growth on 3 different concentrations of BgAtri **(A)** and BgA II (**B**). The experiment was carried out in triplicates. Underlying data can be found in S1 Data. HPAEC-PAD analysis of supernatant from *R*. *gnavus* E1 growth on 1 mM BgAtri **(C)** and *R*. *gnavus* ATCC 29149 growth on 1 mM BgA II **(D)**. The corresponding growth curves are shown in the bottom right of each panel. BgA II, blood group A tetrasaccharide type II; BgAtri, BgA trisaccharide; HPAEC-PAD, high-pH anion exchange chromatography with pulsed amperometric detection.
(TIF)

S13 Fig Enzymatic activity of recombinant WP_004841212.1 fucosidase on BgAtri and mucin. About 1 μM of enzyme was incubated with BgAtri (0.1 mM) (A) or pPGM (1 mg/mL) (B) in 50 mM citrate buffer (pH 6) at 37°C for 24 h. Reactions were then heated at 95°C for 10 min before centrifugation at 17,000*g*, and supernatants were analysed by HPAEC-PAD. Experiments were done at least in duplicates. BgAtri, BgA trisaccharide; HPAEC-PAD, high-pH anion exchange chromatography with pulsed amperometric detection; pPGM, purified pig gastric mucin.
(TIF)

S1 Data Underlying data. (XLSX)

## Abbreviations

aa: amino acid
BgA II: blood group A tetrasaccharide type II
BgAtri: BgA trisaccharide
Cd: central/catalytic domain
CFG: Consortium for Functional Glycomics
C-term: C-terminal
DSF: differential scanning fluorimetry
EFI-EST: Enzyme Function Initiative-Enzyme Similarity Tool
FN3: fibronectin type 3
Fuc: fucose
FUT2: fucosyltransferase 2
GalNAc: N-acetylgalactosamine
GBLD: galactose-binding-like domain
GH: glycoside hydrolase
GI: gastrointestinal
Glc: glucose
GlcNAc: N-acetylglucosamine
HMO: human milk oligosaccharide
HPAEC-PAD: high-pH anion exchange chromatography with pulsed amperometric detection
ICP-MS: inductively coupled plasma mass spectrometry
IMAC: immobilised metal affinity chromatography
IPTG: isopropyl-β-D-thiogalactopyranoside
ITC: isothermal titration calorimetry
IT-sialidase: intramolecular *trans*-sialidase
LC-FD-MS/MS: liquid chromatography with fluorescence detection and mass spectrometric detection
N-term GBLD: N-terminal galactose-binding-like domain
pPGM: purified pig gastric mucin
RBS: ribosome binding site
RFU: relative fluorescence unit
RT-qPCR: quantitative reverse transcription PCR
SEM: standard error of the mean
SSN: sequence similarity network
STD: saturation transfer difference
STD NMR: saturation transfer difference nuclear magnetic resonance spectroscopy
Tm: melting temperature
