# Supplementary figures and images for: The human gut symbiont Ruminococcus gnavus shows specificity to blood group A antigen during mucin glycan foraging: Implication for niche colonisation in the gastrointestinal tract
Source: PLoS Biol. 2021 Dec 22;19(12):e3001498. doi: 10.1371/journal.pbio.3001498 (PMC8730463; doi:10.1371/journal.pbio.3001498)

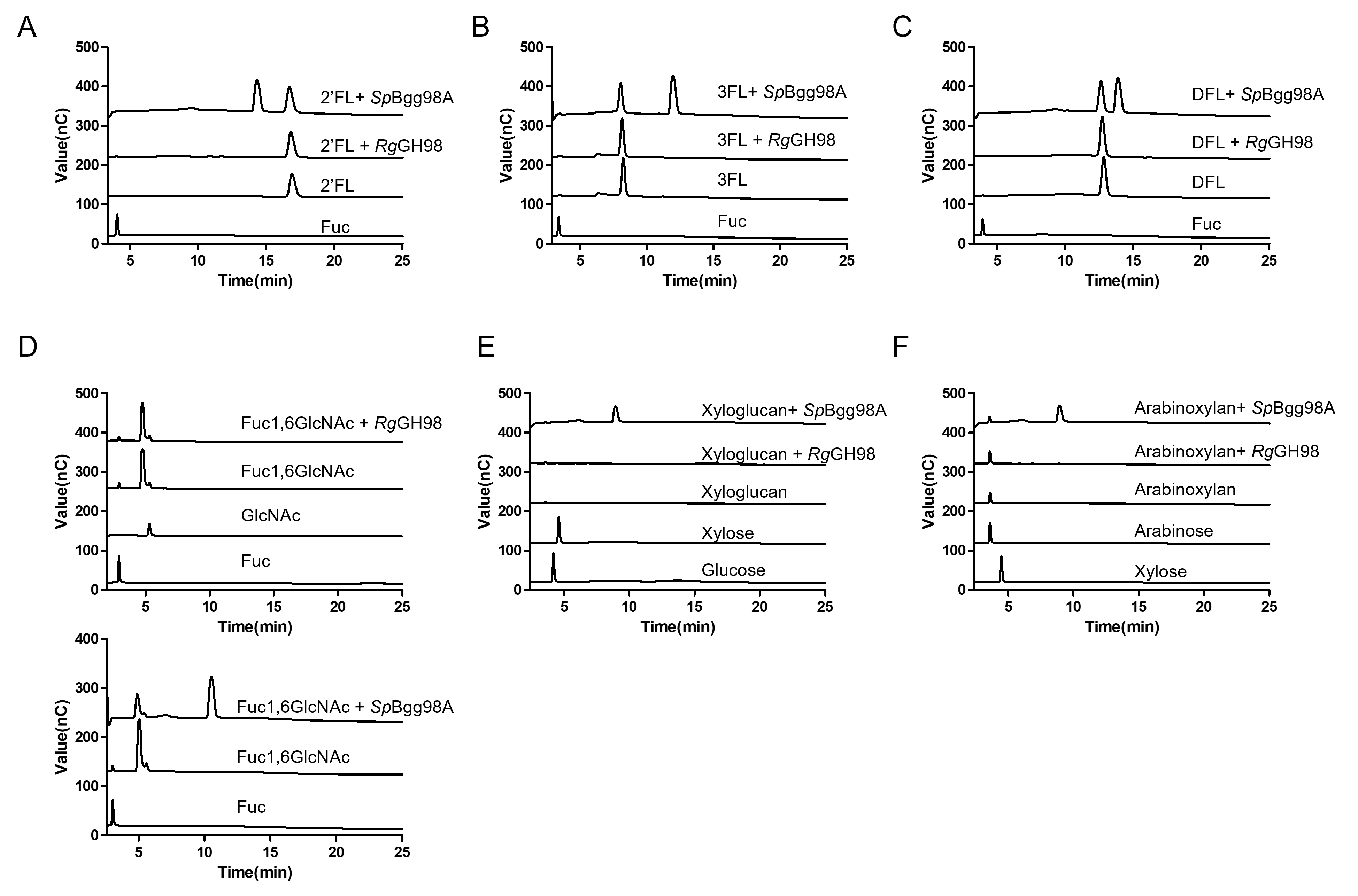

Supplement: S1 Fig — 2′FL (A), 3FL (B), DFL (C), Fucα1,6GlcNAc (D, the upper panel is with RgGH98 and the lower panel is with SpGH98), xyloglucan (E), arabinoxylan (F). DFL, difucosyllactose; Fuc, fucose; HPAEC-PAD, high-pH anion exchange chromatography with pulsed amperometric detection; 2′FL, 2′-fucosyllactose; 3FL, 3-fucosyllactose. (TIF) [file pbio.3001498.s007.tif]

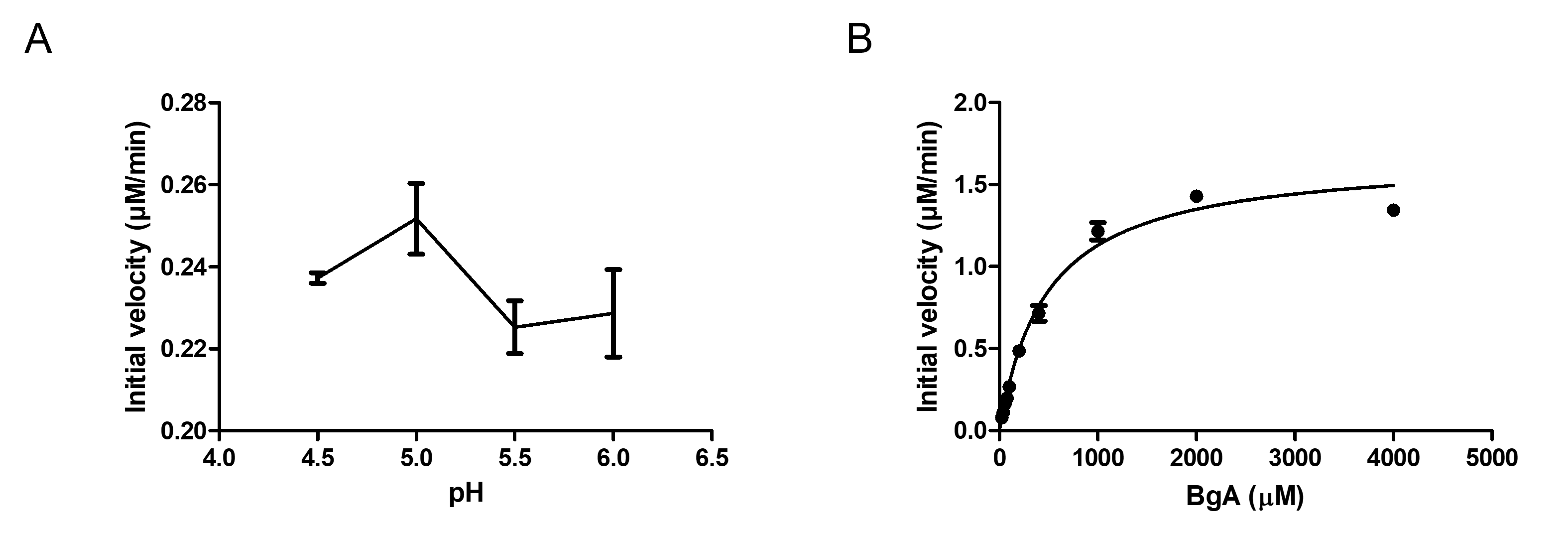

Supplement: S2 Fig — (A) Optimal pH analysis of RgGH98. The initial velocity against BgA II (100 μM) was measured at different pH values in 50 mM citrate buffer with the recombinant enzyme (10 μM) at 37°C. (B) Michaelis–Menten curve of RgGH98 towards BgA II. The experiment was carried out in duplicates for optimal pH analysis and triplicates for Michaelis–Menten curve. BgA II, blood group A tetrasaccharide type II. (TIF) [file pbio.3001498.s008.tif]

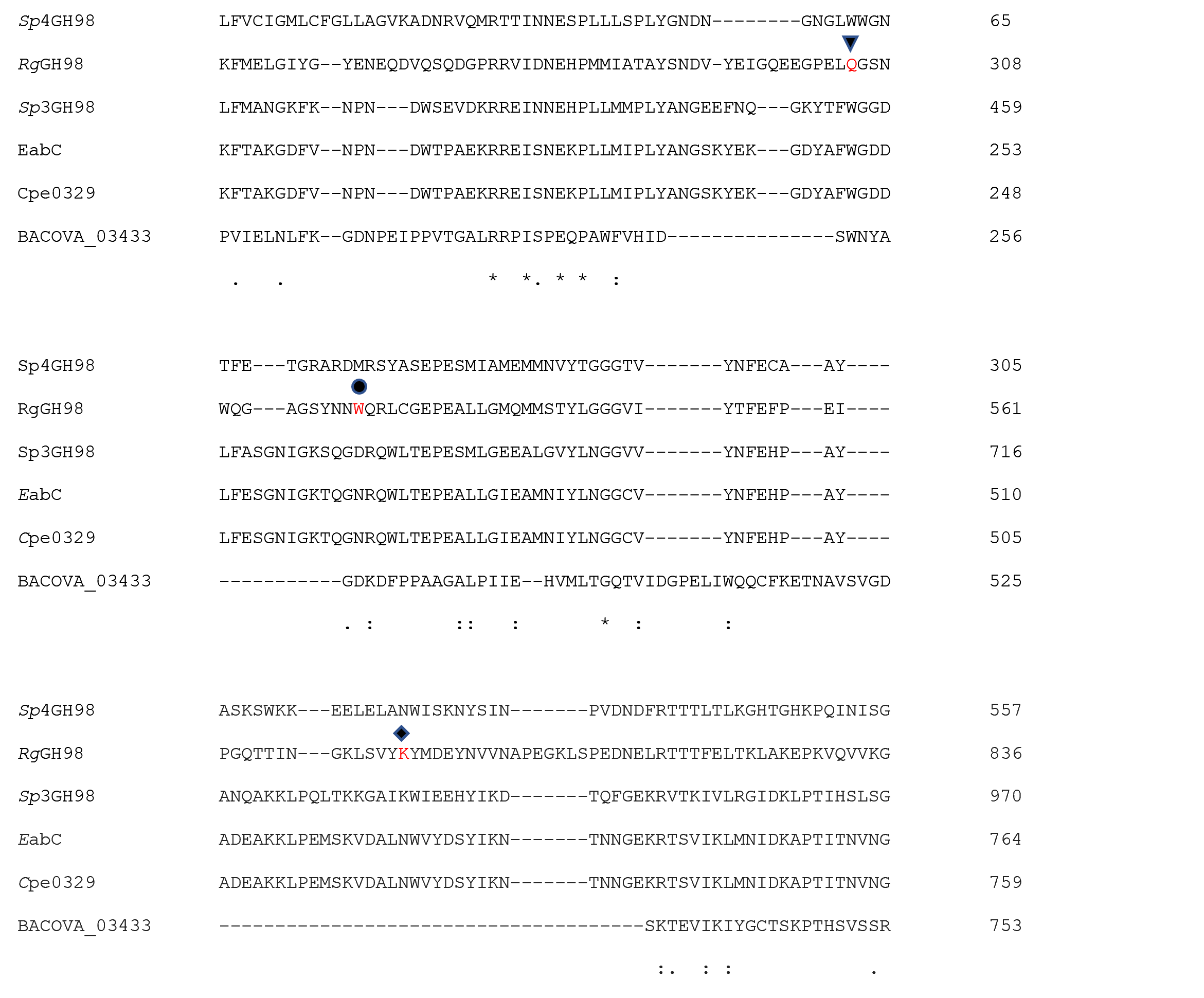

Supplement: S3 Fig — Black triangle, Q305; circle, W528; diamond, K788. (TIF) [file pbio.3001498.s009.tif]

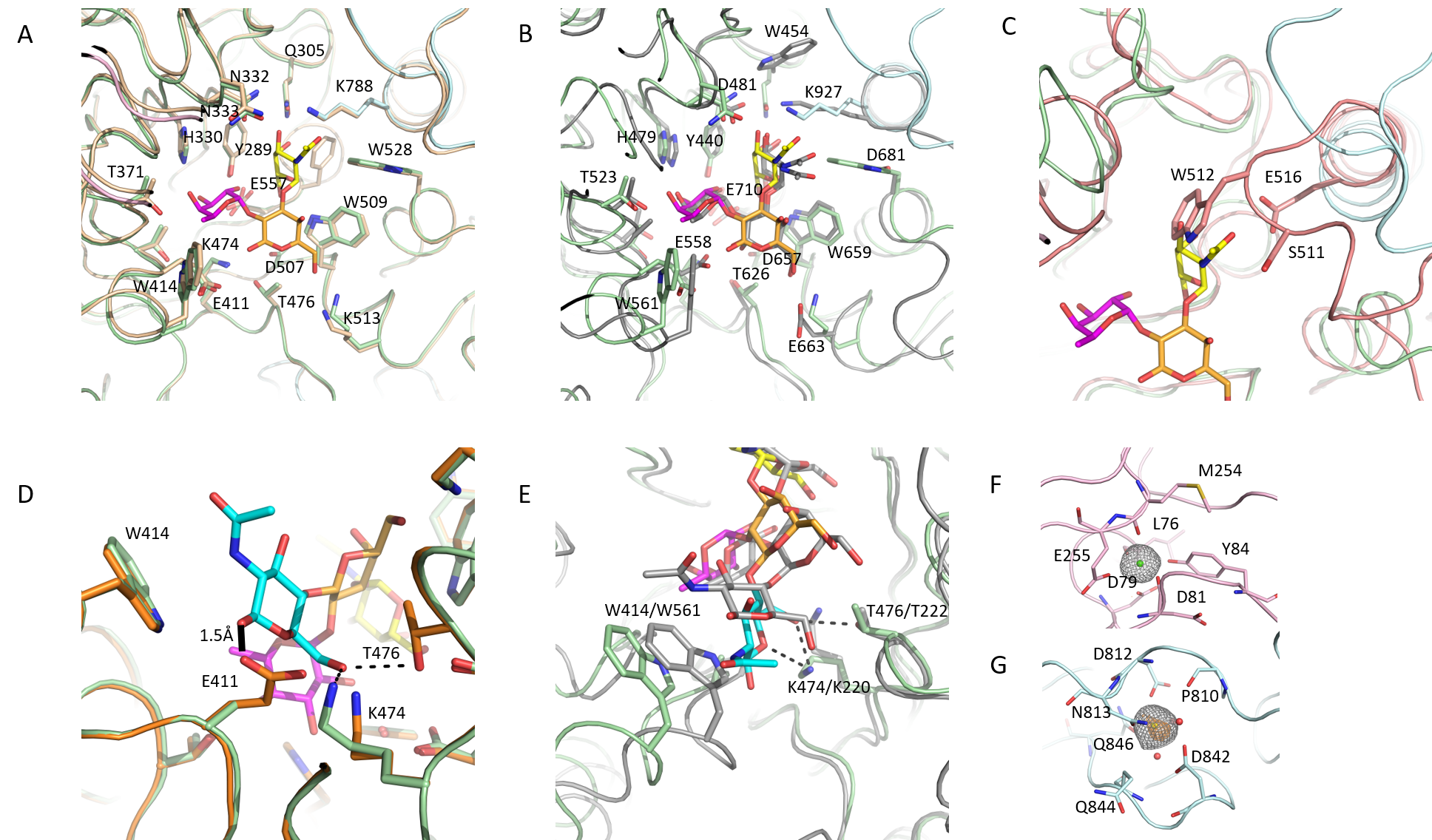

Supplement: S4 Fig — (A) RgGH98 apo structure (wheat) aligned to RgGH98 bound to BgAtri (Cd in green, and C-term domain in light cyan) bound structure. Fuc is coloured in pink, galactose in orange, and GalNAc in yellow. Amino acid identifiers refer to RgGH98. (B) RgGH98 bound to BgAtri aligned to Sp3GH98 (grey) bound to the same carbohydrate. Amino acid identifiers refer to Sp3GH98. (C) RgGH98 bound to BgAtri aligned to Sp4GH98 (pink). Amino acid identifiers to Sp4GH98. The absence of the Trp 512 loop present in Sp4GH98 allows binding of BgA II by RgGH98. (D) RgGH98 bound to BgAtri (orange) aligned to RgGH98 E411A mutant (green) bound to BgA II, highlighting steric clash between BgAtri bound Glu411 rotamer and BgA II. For clarity, only the tetrasaccharide carbohydrate is shown. (E) RgGH98 E411A mutant (green) bound to BgA II aligned to Sp3GH98 (grey) bound to BgA II to highlight differing GlcNAc positioning. First residue number refers to RgGH98 and second to Sp3GH98. (F) Magnesium and (G) calcium binding sites as observed in the RgGH98 E411A with BgA II bound crystal structure. Fo-Fc (grey mesh) and anomolous difference omit maps are shown for both ions, with grey mesh for the Fo-Fc and orange mesh for the anomalous signal. Confirming that only one site is occupied by calcium. BgA II, blood group A tetrasaccharide type II; BgAtri, BgA trisaccharide; Cd, central/catalytic domain; C-term, C-terminal; Fuc, fucose; GalNAc, N-acetylgalactosamine; GlcNAc, N-acetylglucosamine. (TIF) [file pbio.3001498.s010.tif]

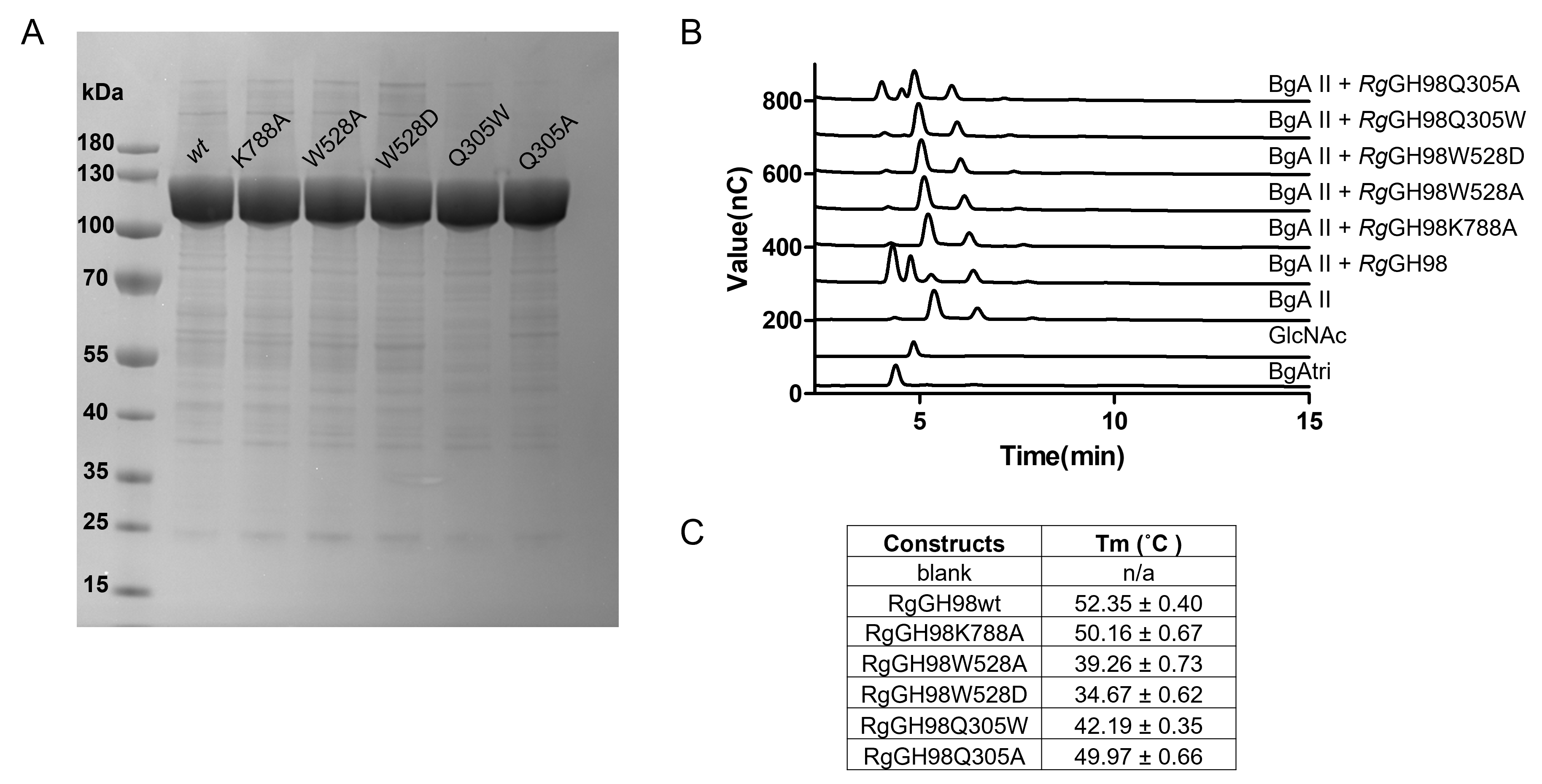

Supplement: S5 Fig — (A). SDS-PAGE analysis of purified RgGH98 wt and specificity mutants. (B) HPAEC-PAD of RgGH98 wt and mutants. Around 10 μM of recombinant enzyme was incubated with BgA II (0.1 mM) in 50 mM citrate buffer (pH 5) at 37°C for 24 h. Reactions were then heated at 95°C for 10 min before centrifugation at 16,000g, and supernatants were analysed by HPAEC-PAD. Experiments were done in triplicates. (C) DSF analysis of RgGH98 wt and mutants. BgA II, blood group A tetrasaccharide type II; DSF, differential scanning fluorimetry; GlcNAc, N-acetylglucosamine; HPAEC-PAD, high-pH anion exchange chromatography with pulsed amperometric detection; wt, wild-type. (TIF) [file pbio.3001498.s011.tif]

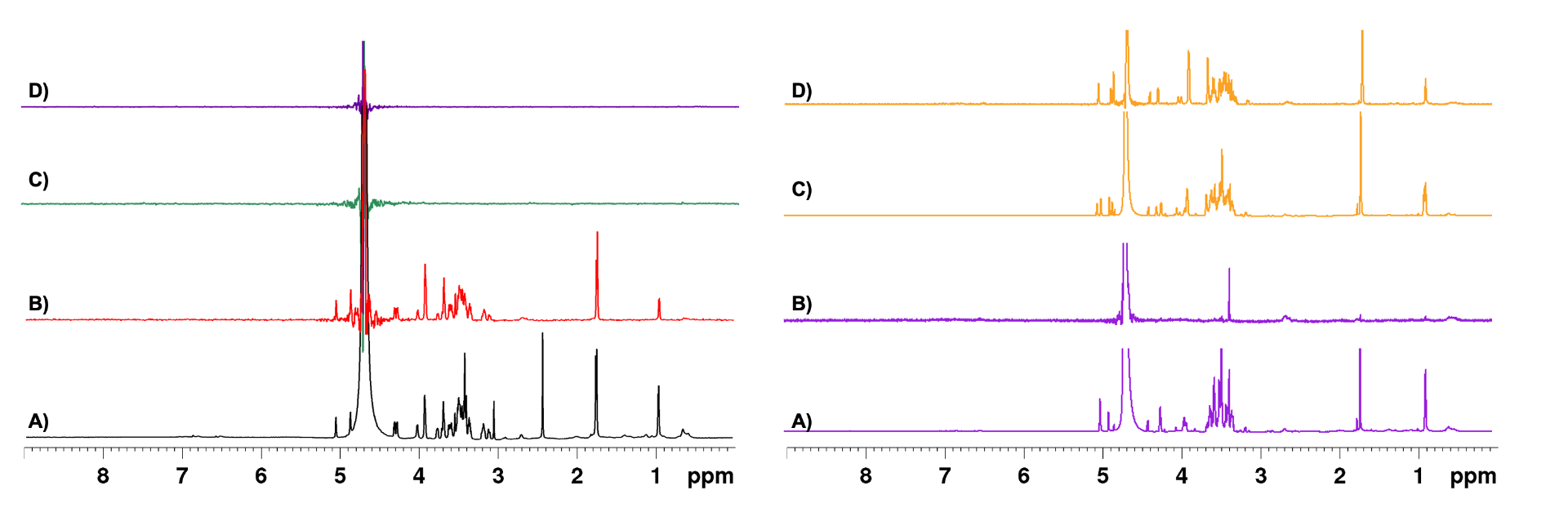

Supplement: S6 Fig — STD NMR binding experiments were performed at 2-s saturation time, with selective protein irradiation at 0.0 ppm. (Left panel) (A) Reference spectrum: BgA II/RgGH98 E411A sample. (B) STD NMR spectrum of BgA II/RgGH98 E411A. (C) STD NMR spectrum of BgA II/RgGH98 N-term GBLD (no binding detected). (D) STD NMR spectrum of BgA II/ RgGH98 C-term (no binding detected). Ligand chemical shifts assignments in S5 Table. (Right panel) (A) Reference spectrum: BgH/RgGH98 E411A sample. (B) STD NMR spectrum of BgH/RgGH98 E411A (no binding detected). (C) Reference spectrum: BgH and BgA II/RgGH98 E411A sample (addition of BgA II to the sample in C). (D) STD NMR spectrum of BgH and BgA II/RgGH98 E411A sample (binding from BgA detected). BgA II, blood group A tetrasaccharide type II; C-term, C-terminal; N-term GBLD, N-terminal galactose-binding-like domain; STD NMR, saturation transfer difference nuclear magnetic resonance spectroscopy. (TIF) [file pbio.3001498.s012.tif]

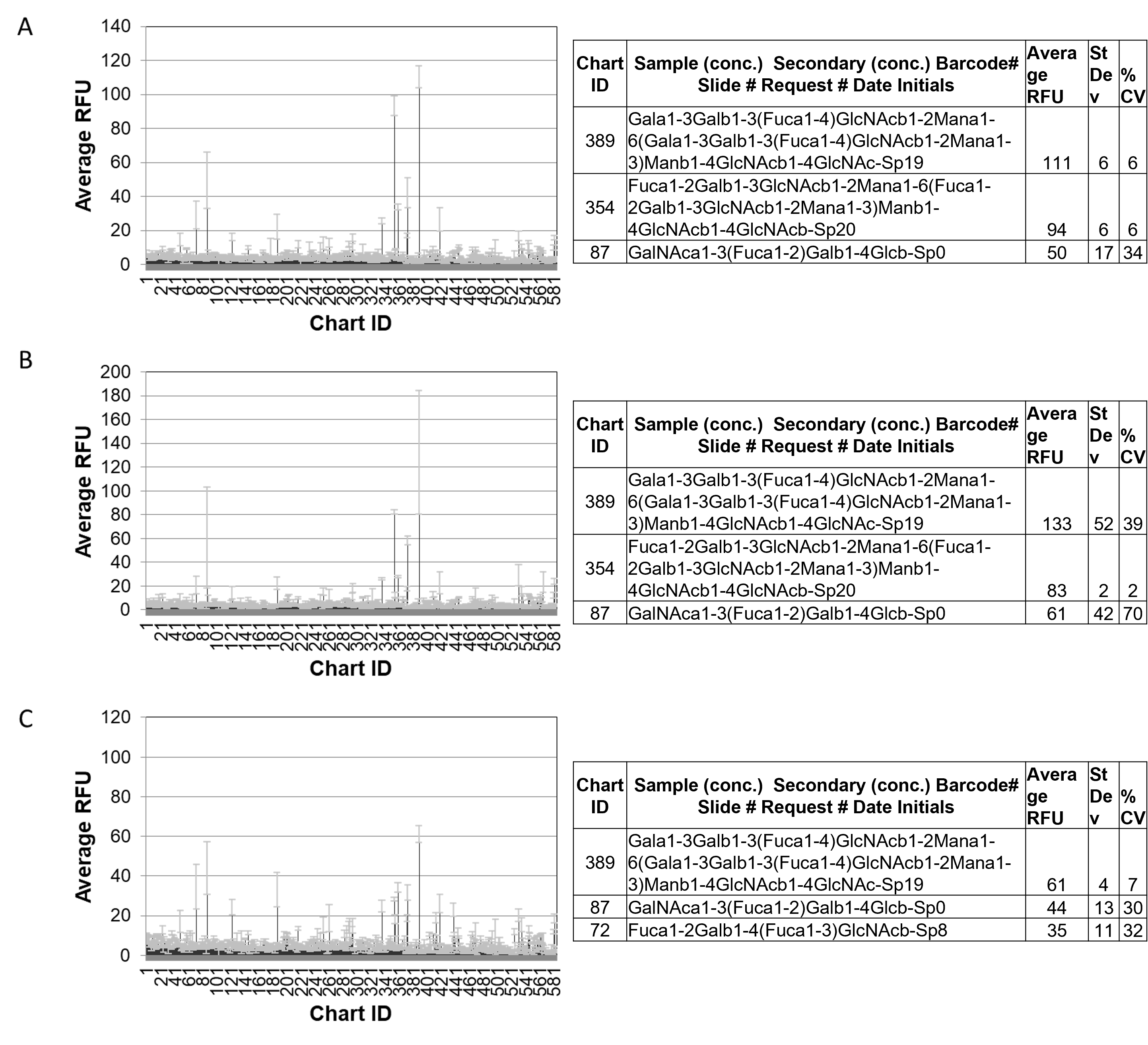

Supplement: S7 Fig — The recombinant proteins (A) RgGH98 E411A, (B) N-term GBLD, and (C) C-term were screened on the CFG glycan array in 6 replicates. The highest and lowest point from each set of 6 replicates has been removed so the average is of 4 values rather than 6. The panels on the left show the overall binding events of RgGH98 variants against 585 ligands. The panels on the right list the glycan compositions of the top 3 hits. Underlying data can be found in S1 Data. CFG, Consortium for Functional Glycomics; C-term, C-terminal; N-term GBLD, N-terminal galactose-binding-like domain; RFU, relative fluorescence unit. (TIF) [file pbio.3001498.s013.tif]

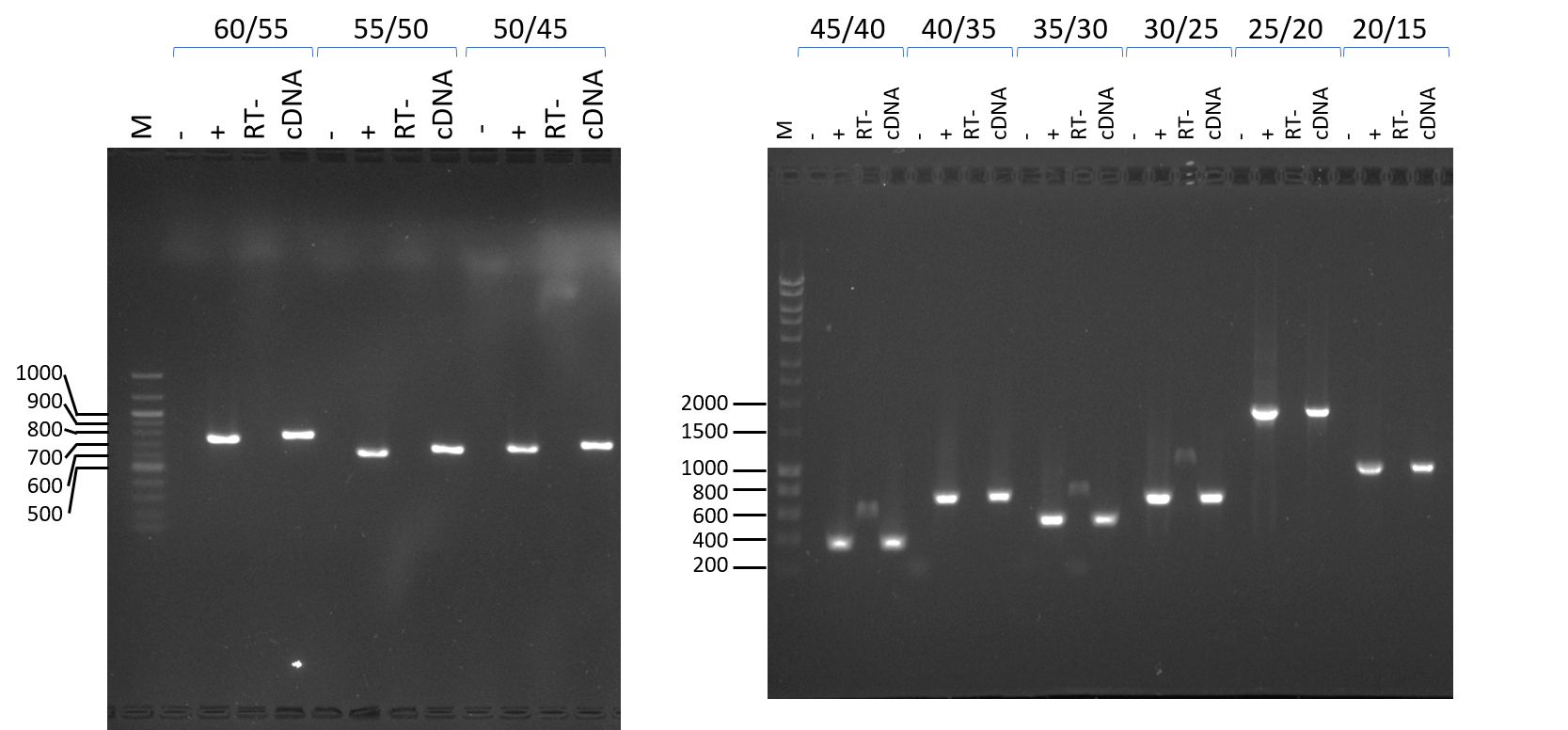

Supplement: S8 Fig — A 2-step RT-PCR was performed on RNA extracted from R. gnavus ATCC 29149 grown on BgA II using primers targeting every intergenic region between genes RGna_RS10360 and RGna_RS10315, and the PCR products analysed by electrophoresis on agarose gel. Intergenic region between RGna_RS103XX and RGna_RS103YY is labelled XX/YY on the gel. PCR from RT negative control (RT−) was performed to confirm the absence of genomic DNA contamination of the RNA sample prior to RT. PCR negative (−) and positive (+) controls were carried out with water or ATCC 29149 genomic DNA as template, respectively. The sequences of the primers are provided in S4 Table. M, DNA ladder size marker (with increments indicated in base pairs). RT-PCR, reverse transcription PCR. (TIF) [file pbio.3001498.s014.tif]

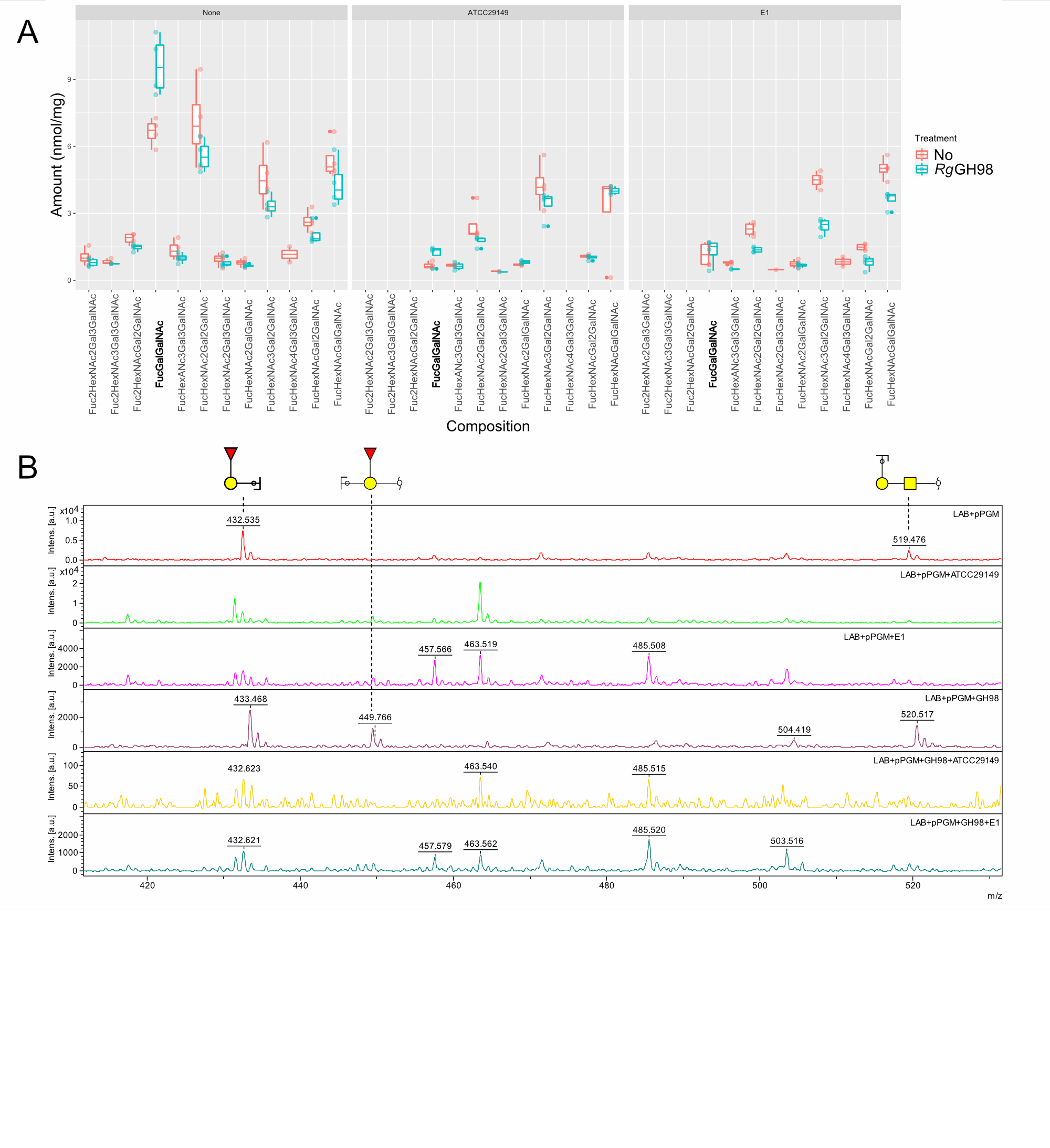

Supplement: S9 Fig — (A) Quantification of fucosylated glycans from growth media supplemented with either untreated (green) or RgGH98-treated (red) pPGM, in the presence or absence of R. gnavus strains. The bold composition “FucGalGalNAc” corresponds to the glycan peak at 708 Da and is indicative of either reduced BgAtri or Fuc-Gal-GalNAc-ol. (B) Fragmentation spectra of the pPGM glycan peak at 708 Da, centred around 470 Da. Fragments characteristic of BgAtri could only be found in the samples with RgGH98-treated pPGM. For glycan analyses, datasets from 2 biological replicates with 2 technical replicates were used. Underlying data can be found in S1 Data. BgAtri, BgA trisaccharide; MS, mass spectrometry; pPGM, purified pig gastric mucin. (TIF) [file pbio.3001498.s015.tif]

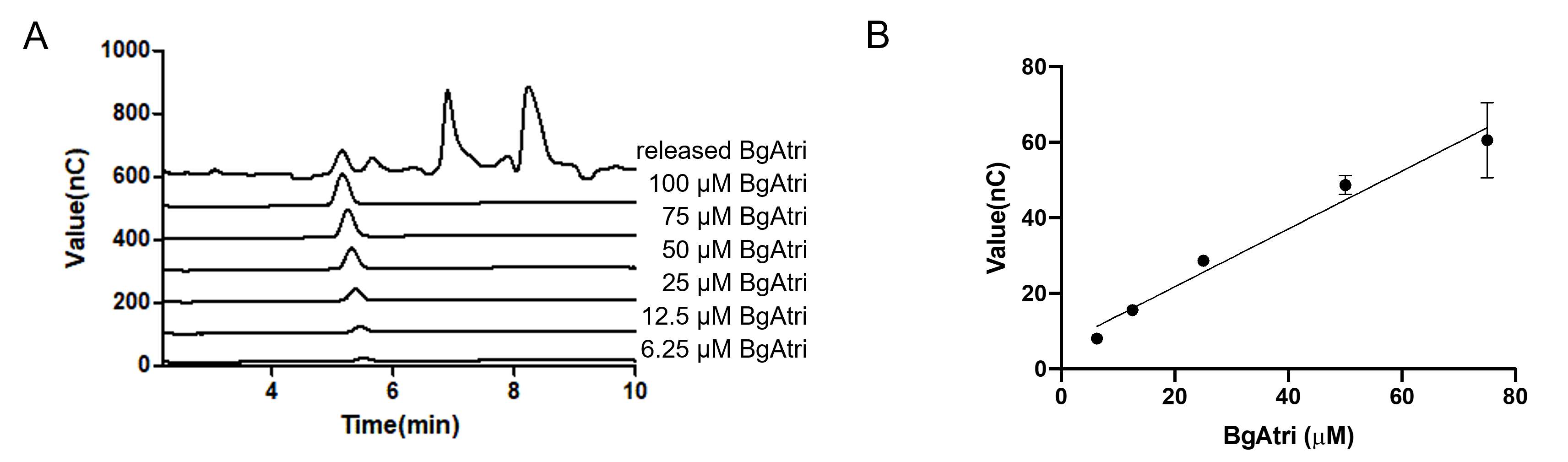

Supplement: S10 Fig — (A) Released BgAtri from the medium prior growth assays along with standards. The experiment was carried out in 3 biological replicates (B) Standard curve was performed in triplicates. The amount of BgAtri released was calculated to be 66.9 μmol/L as average of 4 replicates (67.4 μmol/L, 65.7 μmol/L, 74.1 μmol/L, and 60.5 μmol/L). Underlying data can be found in S1 Data. BgAtri, BgA trisaccharide; HPAEC-PAD, high-pH anion exchange chromatography with pulsed amperometric detection. (TIF) [file pbio.3001498.s016.tif]

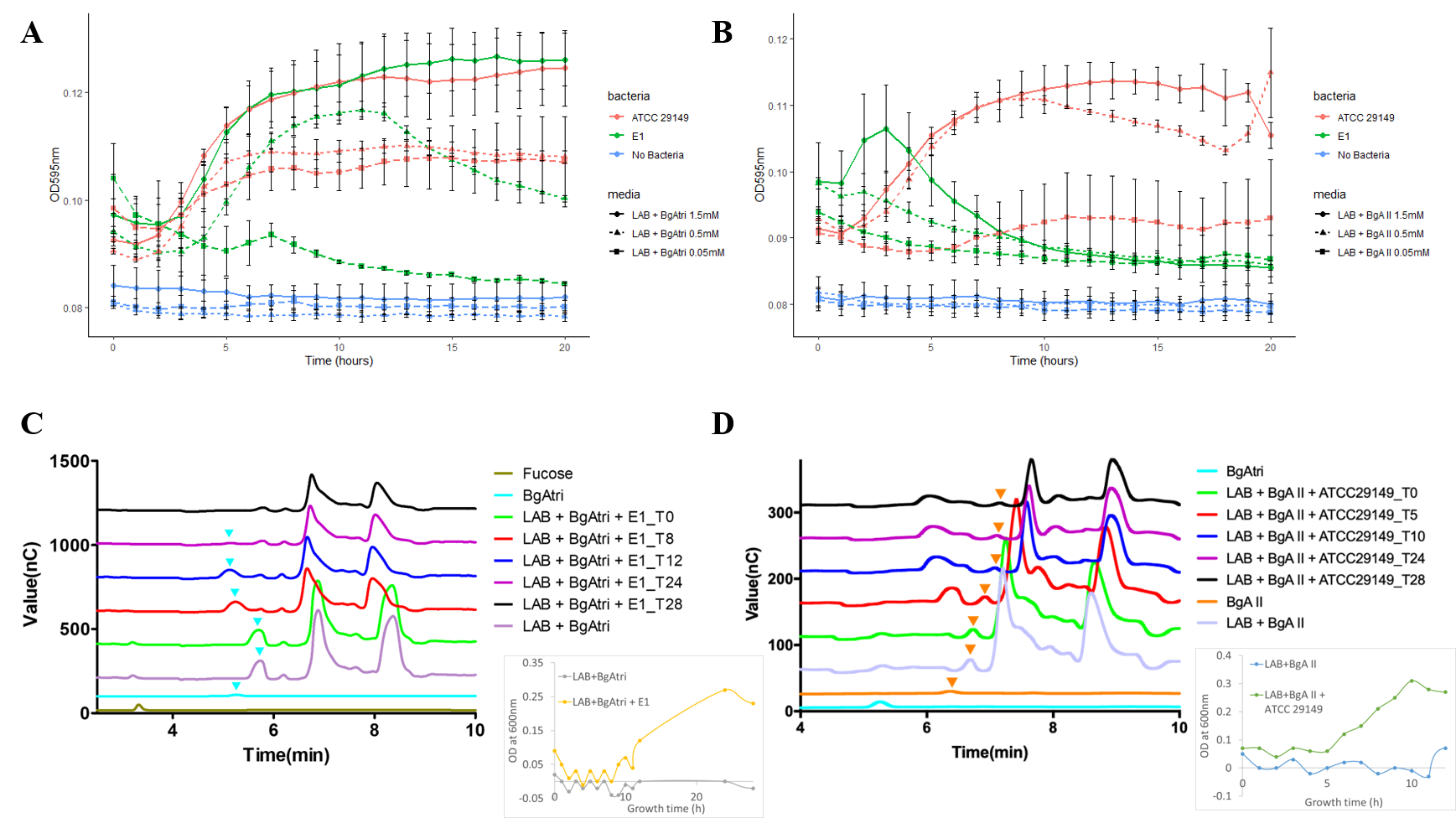

Supplement: S12 Fig — R. gnavus E1 and R. gnavus ATCC 29149 growth on 3 different concentrations of BgAtri (A) and BgA II (B). The experiment was carried out in triplicates. Underlying data can be found in S1 Data. HPAEC-PAD analysis of supernatant from R. gnavus E1 growth on 1 mM BgAtri (C) and R. gnavus ATCC 29149 growth on 1 mM BgA II (D). The corresponding growth curves are shown in the bottom right of each panel. BgA II, blood group A tetrasaccharide type II; BgAtri, BgA trisaccharide; HPAEC-PAD, high-pH anion exchange chromatography with pulsed amperometric detection. (TIF) [file pbio.3001498.s018.tif]

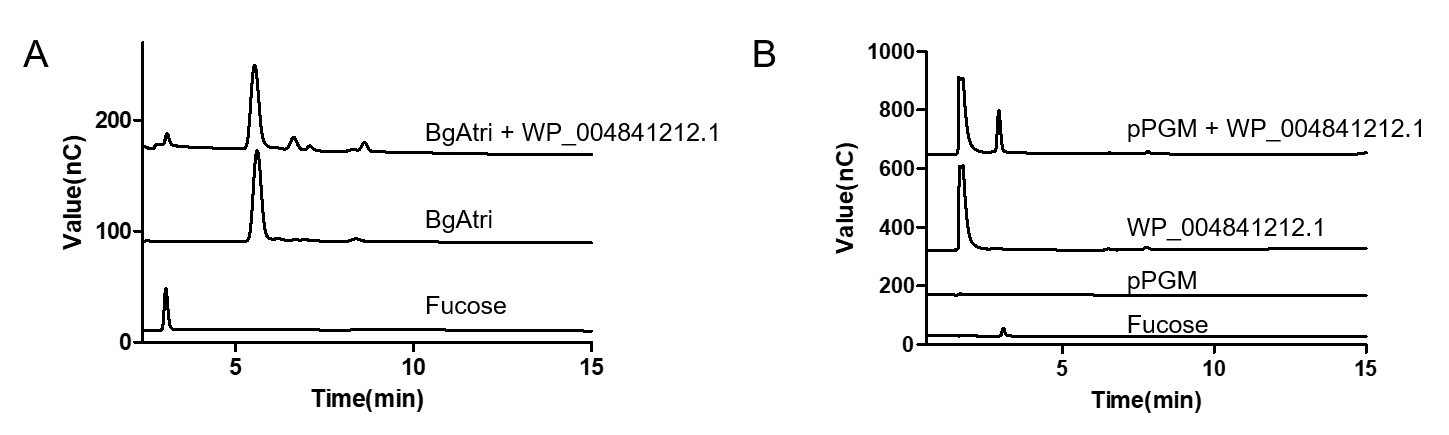

Supplement: S13 Fig — About 1 μM of enzyme was incubated with BgAtri (0.1 mM) (A) or pPGM (1 mg/mL) (B) in 50 mM citrate buffer (pH 6) at 37°C for 24 h. Reactions were then heated at 95°C for 10 min before centrifugation at 17,000g, and supernatants were analysed by HPAEC-PAD. Experiments were done at least in duplicates. BgAtri, BgA trisaccharide; HPAEC-PAD, high-pH anion exchange chromatography with pulsed amperometric detection; pPGM, purified pig gastric mucin. (TIF) [file pbio.3001498.s019.tif]
